# Fic Proteins Inhibit the Activity of Topoisomerase IV by AMPylation in Diverse Bacteria

**DOI:** 10.3389/fmicb.2020.02084

**Published:** 2020-08-26

**Authors:** Can-Hua Lu, Alix McCloskey, Fu-Rong Chen, Ernesto S. Nakayasu, Li-Qun Zhang, Zhao-Qing Luo

**Affiliations:** ^1^Yunnan Academy of Tobacco Agriculture Science, Kunming, China; ^2^Department of Plant Pathology and MOA Key Lab of Pest Monitoring and Green Management, College of Plant Protection, China Agricultural University, Beijing, China; ^3^Department of Biological Sciences, Purdue University, West Lafayette, IN, United States; ^4^Biological Science Division, Pacific Northwest National Laboratory, Richland, WA, United States

**Keywords:** post-translational modification, toxin-antitoxin, AMPylation, filamentation induced by cAMP, DNA gyrase, topoisomerase IV, cell filamentation, DNA replication

## Abstract

The Fic (filamentation induced by cyclic AMP) domain is a widely distributed motif with a conserved sequence of HPFx[D/E]GN[G/K]R, some of which regulate cellular activity by catalyzing the transfer of the AMP moiety from ATP to protein substrates. Some Fic proteins, including Fic-1 from the soil bacterium *Pseudomonas fluorescens* strain 2P24, have been shown to inhibit bacterial DNA replication by AMPylating the subunit B of DNA gyrase (GyrB), but the biochemical activity and cellular target of most Fic proteins remain unknown. Here, we report that Fic-2, which is another Fic protein from strain 2P24 and Fic-1 AMPylate the topoisomerase IV ParE at Tyr^109^. We also examined Fic proteins from several phylogenetically diverse bacteria and found that those from *Yersinia pseudotuberculosis* and *Staphylococcus aureus* AMPylate ParE and GrlB, the counterpart of ParE in Gram-positive bacteria, respectively. Modification by Fic-1 of *P. fluorescens* and FicY of *Y. pseudotuberculosis* inhibits the relaxation activity of topoisomerase IV. Consistent with the inhibition of ParE activity, ectopic expression of these Fic proteins causes cell filamentation akin to the canonical *par* phenotype in which nucleoids are assembled in the center of elongated cells, a process accompanied by the induction of the SOS response. Our results establish that Fic proteins from diverse bacterial species regulate chromosome division and cell separation in bacteria by targeting ParE.

## Introduction

Fic proteins are a family of proteins that harbor the conserved Fic motif (HPFx[D/E]GN[G/K]R). Members of this protein family are found in both prokaryotes and eukaryotes (Kinch et al., 2009; Worby et al., 2009). The majority of characterized Fic proteins catalyze a reversible adenylylation/AMPylation reaction in which the adenosine monophosphate (AMP) moiety is transferred from adenosine triphosphate (ATP) to specific protein targets. This covalent modification induced by Fic proteins typically requires an invariant histidine residue within the core Fic motif (Worby et al., 2009; Yarbrough et al., 2009). In addition to AMPylation, Fic proteins are capable of catalyzing such modifications as UMPylation (Feng et al., 2012), phosphorylcholination (Mukherjee et al., 2011; Tan et al., 2011) and phosphorylation (Castro-Roa et al., 2013; Cruz et al., 2014). The activity of Fic proteins is strictly regulated by a variety of mechanisms, including auto-AMPylation, oligomerization, inter- or intra-molecular inhibition, inhibitory domain-independent inhibition and de-modification by specific enzymes (Tan and Luo, 2011; Tan et al., 2011; Engel et al., 2012; Dedic et al., 2016; Lu et al., 2016; Stanger et al., 2016; Sprenger et al., 2017). In particular, HYPE (also known as FICD) from humans is a bi-functional enzyme that possesses both AMPylation and de-AMPylation activities to regulate the activity of the chaperone BiP in the endoplasmic reticulum (Grammel et al., 2011; Rahman et al., 2012; Ham et al., 2014; Sanyal et al., 2015; Casey et al., 2017; Preissler et al., 2017; Moehlman et al., 2018).

The cellular targets of Fic proteins are highly diverse, ranging from proteins involved in immunity, which are attacked by Fic effectors from pathogens of mammalian or plant hosts (Worby et al., 2009; Yarbrough et al., 2009) to proteins involved in protein folding such as the chaperone BiP (Sanyal et al., 2015), core histones (H2 and H3), translation elongation factors (Castro-Roa et al., 2013; Cruz et al., 2014), and bacterial type IIA family of topoisomerases (GyrB and ParE) (Harms et al., 2015; Lu et al., 2016; Stanger et al., 2016). In most cases, the post-translational modification induced by Fic proteins leads to inhibition of target protein activity (Truttmann and Ploegh, 2017).

The Fic protein VbhT from the bacterial pathogen *Bartonella schoenbuchii* strain R1 modifies both GyrB and ParE by AMPylation (Harms et al., 2015; Siamer and Dehio, 2015). Strain 2P24 of the soil bacterium *Pseudomonas fluorescens* used for biological control against plant diseases (Wei and Zhang, 2006; Wu et al., 2010; Zhang et al., 2014) harbors multiple Fic proteins. Among them, Fic-1 interferes with DNA replication by AMPylating the conserved Tyr^111^ of *P. fluorescens* GyrB (PfGyrB), leading to inhibition of its ATPase activity (Lu et al., 2016).

In an effort to identify additional targets of Fic-1, we found that it recognizes and AMPylates ParE of *P. fluorescens* (PfParE) at Tyr^109^, a residue that is corresponding to Tyr^105^ of ParE from *Escherichia coli*. We also found that Fic-2, another Fic protein from *P. fluorescens* modifies ParE. We extended our study by examining nine Fic proteins from several phylogenetically diverse bacterial species for their ability to modify GyrB or ParE and found that Fic-2 of *P. fluorescens*, FicY of *Yersinia pseudotuberculosis* and Sa1560 of *Staphylococcus aureus* function to modify ParE or GrlB, the counterpart of ParE in Gram-positive bacteria by AMPylation. We further showed that the modification inhibits ParE’s ability to relax supercoiled DNA. Finally, heterologous expression of Fic-2_E56G_ which is a constitutively active mutant of Fic-2, FicY, PA1366, and Fic-1 in *E. coli* caused cell filamentation and the induction of the SOS response. Our results suggest that the ParE subunit of Topo IV appears to be a common target for Fic proteins in a diverse set of bacteria, which play a role in the regulation of DNA replication under certain conditions.

## Materials and Methods

### Bacterial Strains, Plasmids, and Media

The bacterial strains and plasmids used in this study are listed in Supplementary Table S1. All oligonucleotide primers are listed in Supplementary Table S2. *E. coli* strains DH5α and BL21(DE3) were grown in Luria-Bertani (LB) medium at 37°C. *P. fluorescens* strain 2P24, *Y. pseudotuberculosis* strain YPIII and *E. coli* strain BTH101 were grown at 30°C in LB medium. For induction of proteins of interest, overnight cultures grown in LB supplemented with 0.2% glucose and the appropriate concentration of antibiotics were washed with distilled water once and diluted five-fold using Agrobacterium mannitol (ABM) minimal medium with 0.5% of glycerol as the carbon source and 0.2% of arabinose as the inducer. When needed, media were supplemented with antibiotics at the following concentrations: ampicillin (100 μg/ml), kanamycin (50 μg/ml), gentamicin (10 μg/ml), chloramphenicol (20 μg/ml), and tetracycline (5 μg/ml).

### Bacterial Two-Hybrid

The protocol used for bacteria two-hybrid assays was performed as described previously (Lu et al., 2016). The genes of interest were cloned onto pKT25 or pUT18C, and the interaction between Fic-1 and ParE was assayed on LB plates supplemented with X-gal. The strength of the interactions was quantified by measuring the activity of galactosidase (Miller, 1972). Briefly, the OD_600_ of cultures grown in LB broth with the appropriate concentration of kanamycin and ampicillin for 24 h at 30°C was measured. 0.1 ml of samples were added to 0.9 ml of Z-buffer (60 mM Na_2_HPO_4_⋅12H_2_O, 40 mM NaH_2_PO4⋅2H_2_O, 1 mM MgSO_4_⋅7H_2_O, 10 mM KCl and 50 mM β-mercaptoethanol, pH 7.0), 40 μl of chloroform and 20 μl of 0.1% SDS were also added. Mixtures were incubated at 30°C for 15 min, then 0.2 ml of 4 mg/L ortho-nitrophenyl-β-galactoside (ONPG) was added to start the reactions, which were then stopped by adding 0.2 ml of Na_2_CO_3_ until the color of the solutions became light yellow. If the color change was not detected, all reactions were stopped 30 min after the addition of ONPG. After centrifugation at 12,000 rpm for 5 min, OD_420_ of the supernatants was measured by a spectrophotometer. Each sample set was measured in triplicate. β-galactosidase activity was calculated by the equation: 1U = (OD_420_ × 1000)/(OD_600_ × Volume × Time), in which the unit of volume is ml and time is min.

### Plasmids and Strains Construction

Putative Fic proteins from *P. fluorescens* 2P24, *P. aeruginosa* PAO1, *Y. pseudotuberculosis* YPIII, *S. aureus* USA300 and *Streptococcus pneumoniae* D39 each was cloned into the expression vector pETSUMO as *Bam*HI/*Sal*I fragments. The *fic* gene from *Mycobacterium tuberculosis* CDC1551 was inserted into pET22b(+) or pIADL16 as a *Nde*I/*Sal*I fragment to produce His_6_-tagged or His_6_-MBP (Maltose binding protein)-tagged proteins, respectively.

The gene for the DNA gyrase subunit B (*gyrB*) and its counterpart, topoisomerase IV subunit B (*parE* or *grlB*) for relevant bacteria were inserted into pETSUMO to produce His_6_-tagged proteins. For *M. tuberculosis* CDC1551, the only *gyrB* was cloned as it does not have a *parE* gene.

For expression of Fic-2 in *P. fluorescens* 2P24, pCL008 was used as described previously (Lu et al., 2016). To test its effects on cell morphology, the *fic* gene from *Y. pseudotuberculosis* YPIII (Wang et al., 2015) was inserted into pBAD22, its expression was induced by 0.2% arabinose. The integrity of each gene was verified by double-strand sequencing analysis.

### Site-Directed Mutagenesis

We used the QuikChange^®^ mutagenesis kit to introduce mutations into specific sites of genes of interest by the high-fidelity DNA polymerase Pfu Ultra II (Agilent Technologies). The primers were designed by the QuikChange^®^ Primer Design Program (Agilent Technologies).

### Expression and Purification of Proteins

Unless otherwise stated, *E. coli* strain BL21(DE3) was used to express proteins of interest. The bacterial strain was inoculated in 25-ml of L. broth with appropriate antibiotics in a 250-ml flask shaking at 225 rpm for 6 h. Each culture was sub-cultured in 500 ml of L. broth in a 2-L flask until the absorbance at 600 nm reached between 0.6 and 0.8. Isopropyl thio-D-galactopyranoside (IPTG) was then added to a final concentration of 250 μM, and the induction was allowed to proceed for 16 h at 18°C. The cells were harvested by centrifugation at 6,000 rpm for 10 min at 4°C and resuspended in 30 ml cold PBS buffer containing 20 mM imidazole and protease inhibitors benzamidine and phenylmethanesulfonyl fluoride (PMSF) at final concentrations of 5 and 1 μM, respectively. Mixtures were then lysed by sonication. After spinning down the pellets by centrifugation at 12,000 rpm for 15 min twice at 4°C, supernatants were mixed with 2 ml Ni-NTA beads in 50-ml Falcon tubes, Triton X-100 was added to a final concentration of 0.5% and the binding was allowed to proceed at 2 h at 4°C while rotating. The Ni^2+^-NTA beads were loaded into a 30-ml column and beads were washed with 60 ml PBS buffer containing 20 mM imidazole by gravity flow. The bound proteins were eluted with PBS buffer containing 250 mM imidazole. The purity of the protein (normally 2–5 μl) was assessed by SDS-PAGE and Coomassie brilliant blue before dialyzing against 1 L PBS containing 20% (v/v) glycerol and 1 mM β-mercaptoethanol at 4°C for 16 h. Protein concentration was determined by the Bradford assay (Bio-Rad). When needed, the SUMO protease Ulp1 was used to cleave the His_6_-SUMO tag from fusion proteins at 30°C for 3 h.

### *In vitro* AMPylation Assay

The AMPylation assay was performed as described with minor modifications (Tan and Luo, 2011; Tan et al., 2011; Lu et al., 2016). Briefly, 3 μg of purified Fic was incubated with 10 μg His_6_-SUMO-GyrB or His_6_-SUMO-ParE for 45 min at 35°C in 20 μl of reaction buffer containing 25 mM Tris–HCl, pH 7.5, 50 mM NaCl, 3 mM MgCl_2_, 0.5 mM EDTA, and ^32^P-α-ATP (5 μCi) (Perkin Elmer). When needed, 1.5 μg Fic-1-His_6_ was incubated with purified GyrB or ParE proteins. After reactions were stopped by the addition of 5 μl of 5× SDS sample buffer, samples were boiled for 10 min at 100°C and separated on 4–20% gradient SDS-PAGE gels (Bio-Rad) at 120 volts for 80 min. Gels were stained with Coomassie brilliant blue and destained with a solution containing 45% methanol, 10% acetic acid and 45% ddH_2_O. Detection of ^32^P-α-AMP-labeled molecules was done by autoradiography using dried PAGE gels and Biomax MS films (Kodak).

Auto-AMPylation by Fic proteins detected by fluorescence were conducted using the following protocol using the fluorescence ATP analog N6-(6-Amino)-hexyl-ATP-5-FAM (ATP-FAM, JBS-NU-805-5FM, Jena Bioscience). Briefly, ten micrograms of Fic protein were incubated with 5 μM ATP-FAM in 20 μl AMPylation buffer containing 25 mM Tris–HCl, pH 7.5, 50 mM NaCl, 3 mM MgCl_2_ and 0.5 mM EDTA for 1 h at 35°C. The reactions were terminated with the SDS sample buffer and were boiled for 5 min. Proteins were separated on 12% SDS-PAGE gels and fluorescence signals (λ_ex_ = 488 nm, λ_em_ = 526 nm) were measured by using the green channel of an iBright FL1500 Imaging System (Thermo Fisher Scientific).

To test the inhibition of FicY by AntY, a 120 μl master reaction containing 30 μg His_6_-SUMO-FicY was used to set up sub-reactions with different amounts of His_6_-SUMO-AntY. Reactions were incubated at room temperature for 30 min, followed by the addition of 10 μg His_6_-SUMO-ParE into each reaction. A reaction not receiving His_6_-SUMO-AntY was used as a control. After incubation at 35°C for 30 min, the reactions were terminated with the SDS sample buffer. Reaction products were separated on 4–20% prepared SDS-PAGE, and signals were detected by autoradiography with Biomax MS films (Kodak).

### Negative Supercoiling and Relaxation of Plasmid DNA *in vitro*

Supercoiled plasmid DNA was prepared by isolating pHSG399 from *E. coli* strain DH5α. Relaxed plasmid DNA was produced by treating supercoiled pHSG399 with topoisomerase I (NEB) in 1× CutSmart buffer (50 mM KAc, 20 mM Tris–Ac, 10 mM Mg(Ac)_2_, and 100 μg/ml BSA, pH 7.9; NEB).

The negative supercoiling assay was performed in a reaction mixture containing 1× DNA gyrase reaction buffer (35 mM Tris–HCl, pH 7.5 at 25°C, 24 mM KCl, 4 mM MgCl_2_, 2 mM DTT, 1.75 mM ATP, 5 mM spermidine, 0.1 mg/ml BSA, 6.5% Glycerol; NEB), 0.3 μg of relaxed pHSG399 DNA, and appropriate amounts of DNA gyrase. The optimal concentration of gyrase was determined by adding a diluent. For dose-dependent inhibition of DNA gyrase supercoiling activity by Fic proteins, increasing amounts of Fic proteins were pre-incubated with DNA gyrase in a reaction mixture without relaxed DNA at 30°C for 1 h. The reactions were initiated by adding 0.3 μg relaxed pHSG399, incubated for 1 h, and stopped using 20 mM EDTA.

Relaxation of negative supercoils was assayed in a reaction mixture (20 μl) containing 35 mM Tris–HCl, pH 7.5 at 25°C, 24 mM KCl, 4 mM MgCl_2_, 2 mM DTT, 1.75 mM ATP (unless indicated otherwise), 5 mM spermidine, 0.1 mg/ml BSA, 6.5% Glycerol, 0.3 μg of negatively supercoiled pHSG399 DNA, and indicated amounts of Topo IV. The effect of Fic on the relaxation of negative supercoiled DNA by DNA gyrase was determined in reactions without ATP. The optimal concentration of Topo IV from each organism was determined by three-fold dilution of the proteins. When needed, a set amount of Fic proteins was pre-incubated with Topo IV in reaction buffer without DNA at 30°C for 1 h. The reaction was started by adding 0.3 μg negatively supercoiled pHSG399 DNA, continued for 1 h at 30°C, and terminated by EDTA (final concentration of 20 mM). The reactions were analyzed by electrophoresis in 1.2% agarose gels at 2.5 V/cm for 12 h in 50 mM Tris–HCl, pH 8.0 at 25°C, 40 mM NaOAc, and 1 mM EDTA running buffer. DNA agarose gels were stained with 1 μg/ml ethidium bromide for 10 min prior to image acquisition by an iBright FL1500 Imaging System.

### Microscopy

After induction of Fic, ParE and their mutant versions, bacteria cells of *E. coli*, *P. fluorescens* or *Y. pseudotuberculosis* were washed with PBS and fixed with 4% paraformaldehyde following a described protocol (Lu et al., 2016).

### Antibodies and Immunoblotting

SUMO, FicY, and ParE-specific antibodies were raised and purified as described earlier (Lu et al., 2016). The antibody was used at 1:10,000 for immunoblotting. Antibodies against ICDH (Lu et al., 2016), RecA (Santa Cruz Biotechnology), and LexA (Abcam) were used at 1:10,000, 1:3000 and 1:5000, respectively.

For immunoblotting, samples were separated by 8–15% SDS-PAGE, proteins were transferred onto nitrocellulose membranes and the membranes were blocked with 5% nonfat milk in PBS with 0.2% Tween 20 (PBST) for 1 h, the membranes were washed three times with PBST buffer and incubated with the primary antibody for 2 h. After washing, membranes were incubated with a fluorescence-labeled secondary antibody labeled with IRDye 700 or IRDye 800 (Li-COR Biosciences), and the signals were detected with the LI-COR Odyssey Imaging System (Li-COR Biosciences).

### Statistical Analyses

Images of blots were converted into a JPEG file format, then changed the picture mode to “Grayscale”. The intensity of target bands was relatively quantified by using NIH ImageJ (version 1.52q) densitometric software. The same frame was used for all of the protein bands [including LexA, RecA and ICDH (isocitrate dehydrogenase)] and their backgrounds. The pixel density for all data was inverted (255-*X*, where *X* is the value recorded by Image J). For the protein bands and loading controls, express the net value by deducting the inverted background from the inverted band value. The final relative quantification values are the ratio between net band of the samples and that of the loading control. The Student’s *t* test was used to compare the mean levels between the two groups. A *p*-value less than 0.05 was considered statistically significant.

## Results

### Fic-1 AMPylates Topo IV Subunit B (PfParE) at Tyr^109^

**FIGURE 1:**
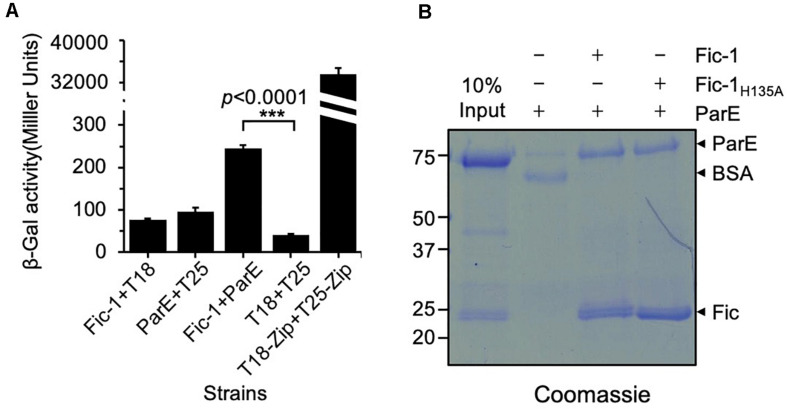
Fic-1 interacts with ParE. **(A)** Fic-1 interacts with PfParE in a bacterial two-hybrid assay. Fic-1 and ParE were fused to the T25 and T18 fragments of the *Bordetella pertussis* adenylate cyclase, respectively. The plasmids pKT25-zip and pUT18C-zip, expressing T25 and T18 fused to a leucine zipper motif that strongly interact, respectively, serve as the positive control. Cells containing the indicated plasmids were grown in LB liquid for 24 h at 30°C. The interactions were quantified by measuring galactosidase activity. Data shown represent the average of three independent experiments. *p*-values were calculated using the Student’s *t*-test. **(B)** Fic-1 interacts with ParE *in vitro*. Fifty micrograms of Fic-1-His_6_ or Fic-1_H135A_-His_6_ was used to coat Affigel-15 beads, control beads were coated with 50 μg BSA. After blocking with 20 mM Tris–HCl, washed beads were incubated with 40 μg ParE for 4 h at 4°C. After extensive washing, bound proteins were separated by SDS-PAGE and detected by Coomassie brilliant blue staining. 10% input was loaded as a reference. Note that the predicted molecular mass of Fic-1-His_6_ is about 23.34 kDa, and His_6_-SUMO-PfParE is 83.33 kDa. Data shown are representative of three experiments with similar results.

We previously showed that Fic-1 (locus tag: C0J56_10235) of *P. fluorescens* strain 2P24 inhibits DNA replication by AMPylating GyrB (Lu et al., 2016). Since ParE is a paralog of GyrB, we examined its interactions with Fic-1. Binding between these two proteins was evident in a bacterial two-hybrid assay as significant galactosidase activity was observed in the *E. coli* strain BTH101 (Karimova et al., 1998) co-expressing Fic-1 and PfParE fused to the T18 and T25 fragment of adenylate cyclase (CyaA), respectively (Figure 1A). Furthermore, PfParE can be co-purified with Fic-1 or Fic-1_H135A_ (mutation of His135 to Ala in the conserved Fic motif) in the pulldown assay (Figure 1B). Thus, Fic-1 detectably interacts with ParE, and its inactive mutant Fic-1_H135A_ retains this binding activity.

**FIGURE 2:**
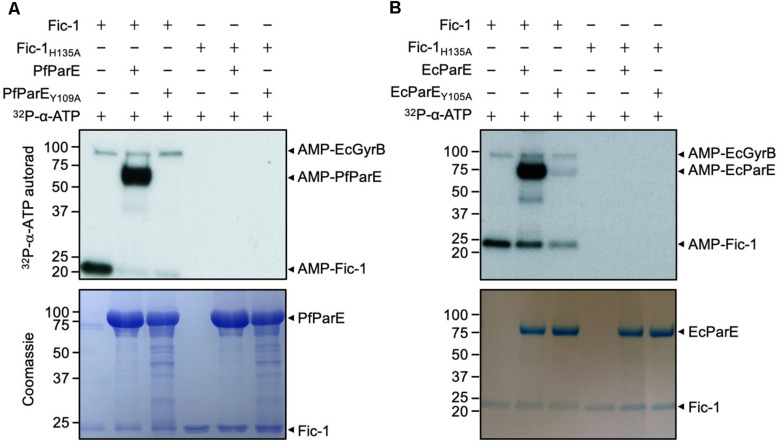
Fic-1 AMPylates ParE of *P. fluorescens* at Tyr^109^ **(A)** and of *E. coli* at Tyr^105^ **(B)**. Fic-1 or Fic-1_H135A_ was incubated with ParE or its mutant at 35°C for 30 min. The reaction was terminated with Laemmli buffer. Modification was detected after SDS-PAGE by autoradiography (upper panel) and Coomassie staining (lower panel). Note the robust AMPylation of PfParE but not PfParE_Y109A_. EcGyrB co-purified with Fic-1 indicated by arrows. Note that the predicted molecular mass of Fic-1-His_6_, EcGyrB and His_6_-SUMO-EcParE is about 23.34, 89.95, and 84.04 kDa, respectively. Autoradiographs of 30 min (**A** upper panel) and 5 min (**B** upper panel) exposure times were shown.

The interaction between ParE and Fic-1 prompted us to determine whether Fic-1 could modify the DNA topoisomerase by AMPylation. We thus incubated Fic-1 and ParE together with ^32^P-α-ATP and examined potential modification by autoradiograph. Our results revealed that ParE from both *P. fluorescens* (PfParE) and *E. coli* (EcParE) can be robustly AMPylated by Fic-1 and such modification completely depended upon the histidine residue essential for its enzymatic activity (Figures 2A,B).

Residue Tyr^109^ in PfParE is conserved in ParE across prokaryotes and is functionally equivalent to Tyr^111^ in PfGyrB, which is the site modified by Fic-1 (Supplementary Figure S1A) (Lu et al., 2016). We therefore speculated that Tyr^109^ is the likely AMPylation site on PfParE. Indeed, a Y109A mutation in this protein rendered it no longer modifiable by Fic-1 (Figure 2A). Mutation of the corresponding residue Tyr^105^ (Supplementary Figure S1A) also abolished the ability of EcParE to be modified by Fic-1 (Figure 2B). In these experiments, a ^32^P-labeled protein with a molecular weight higher than that of ParE was detected (Figure 2). This band likely is endogenous *E. coli* GyrB co-purified with Fic-1 due to the high affinity between these two proteins (Lu et al., 2016). In reactions containing the EcParE_Y105A_ mutant, the signals were still detectable (Figure 2B), which may result from additional modification sites on EcParE.

### Fic Proteins From Diverse Bacteria AMPylate ParE *in vitro*

Fic proteins are present in a large cohort of bacteria, both Gram-positive and Gram-negative, but their cellular targets in most of these microorganisms remain unknown (Veyron et al., 2018). The finding that Fic-1 modified GyrB and ParE prompted us to examine whether these proteins are modified by Fic proteins in several taxonomically diverse bacteria, including the *E. coli* strain K12 (Blattner et al., 1997), *P. aeruginosa* strain PAO1 (Stover et al., 2000), *Mycobacterium tuberculosis* strain CDC1551 (Liu et al., 2016), *Y. pseudotuberculosis* strain YPIII (Johnson et al., 2015), *S. aureus* strain USA300 (Diep et al., 2006), and *S. pneumoniae* strain D39 (Lanie et al., 2007) (Supplementary Figures S1B,C). Some Fic proteins from these species, including EcFic (locus tag: b3361) from *E. coli* strain MG1655, PA1366 from *P. aeruginosa* strain PAO1, MT3743 from *M. tuberculosis* strain CDC1551 and FicY (locus tag: YPK_0608) from *Y. pseudotuberculosis* YPIII, belong to class I Fic. The first three Fic proteins listed above appear to be regulated by a predicted intermolecular inhibitor protein encoded upstream of these Fic genes, whereas the predicted inhibitory motif for FicY is encoded by a downstream, convergent open reading frame that we termed *antY* (Supplementary Figures S1D,E,S2). The predicted Fic-2 (locus tag: C0J56_09765) and Fic-3 (locus tag: C0J56_23350) from *P. fluorescens* strain 2P24, PA0574 (locus tag: PA0574) from *P. aeruginosa* strain PAO1 and Sa1560 (locus tag: SAUSA300_1560) from *S. aureus* strain USA300 belong to class II, and Sp0496 (locus tag: SPD_0496) from *S. pneumoniae* strain D39 is a class III Fic protein (Supplementary Figure S2).

**FIGURE 3:**
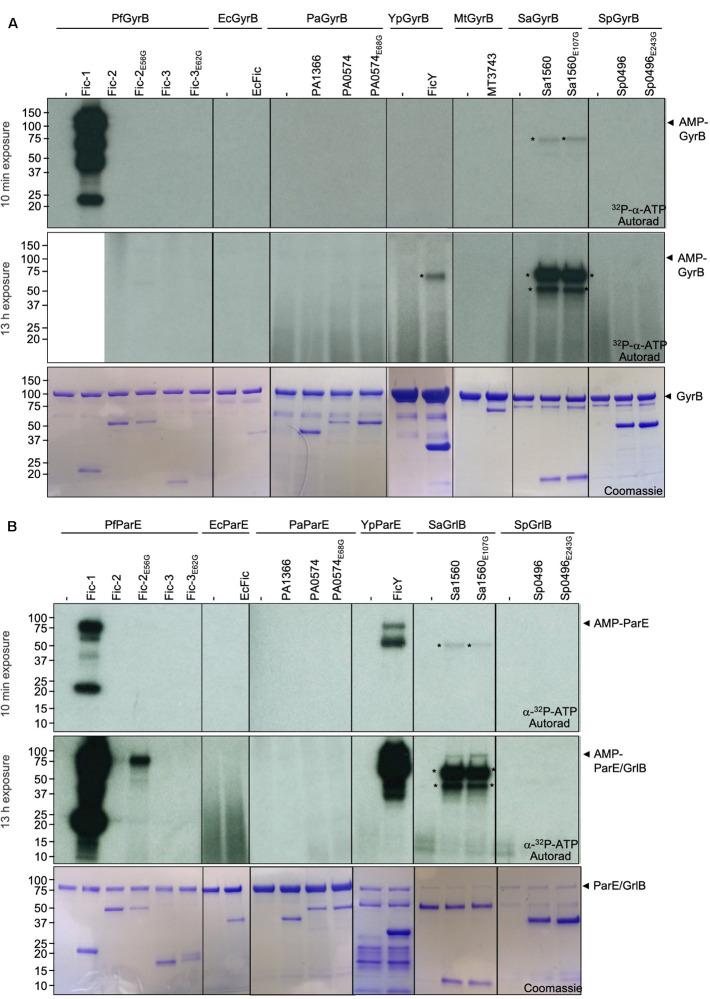
ParE is a preferred substrate for Fic proteins from phylogenetically diverse bacteria. In each case, 10 μg GyrB **(A)** or ParE **(B)** was incubated with 3 μg of Fic proteins or their constitutively active mutants for 45 min. The signals were detected by autoradiography for either 10 min (upper panel) or 13 h (middle panel) exposures (the lane containing Fic-1 and PfGyrB was removed before 13 h exposure) and Coomassie staining (bottom panel). The molecular mass of His_6_-SUMO tagged PfGyrB, EcGyrB, PaGyrB, YpGyrB, MtGyrB, SaGyrB, and SpGyrB is 103.92, 103.75, 103.99, 104.07, 89.12, 86.34, and 86.04 kDa, respectively. The predicted molecular mass of His_6_-SUMO tagged PfParE, EcParE, PaParE, YpParE, SaGrlB, and SpGrlB is 83.33, 84.04, 82.98, 83.79, 88.16, and 85.43 kDa, respectively. The predicted molecular mass of Fic-1-His_6_, MBP-MT3743-His_6_, and His_6_-SUMO tagged Fic-2, Fic-3, EcFic, PA1366, PA0504, FicY, Sa1560, and Sp0496 is 23.34, 67.45, 52.20, 58.48, 36.76, 43.06, 55.76, 37.44, 61.48, and 44.73 kDa, respectively. Radioactive bands those do not corresponding to the substrate were labeled with asterisks. Note that robust AMPylation of GyrB by Fic-1 is seen but no detectable modification occurred for other proteins even after 13 h exposure **(A)**. In contrast, Fic proteins from *P. fluorescens*, *Y. pseudotuberculosis*, and *S. aureus* modified their cognate ParE proteins.

To examine the potential activity of these Fic proteins against GyrB and ParE, we first substituted the glutamate residue within the predicted Fic inhibitory domain (Engel et al., 2012) to create constitutively active mutants Fic-2_E56G_, Fic-3_E62G_, PA0574_E63G_, Sa1560_E107G_, and Sp0496_E243G_. Each Fic protein and its presumably constitutively active mutant were purified from *E. coli* by His_6_ affinity purification. Each of these proteins was incubated in reactions containing ^32^P-α-ATP and their cognate GyrB or ParE. Modification of the potential targets was detected by autoradiograph. In reactions containing GyrB, similar to earlier results, GyrB was robustly AMPylated by Fic-1 (Figure 3A, top panel) (Lu et al., 2016). No signal of AMPylated GyrB was detected in reactions containing Fic proteins from other bacterial species even when the exposure time was extended to 13 h. In addition, a protein of approximately 70 kDa was modified in the reaction containing GyrB and FicY, which may result from an additional target likely co-purified with FicY (Figure 3A, middle panel).

When ParE was used as the substrate, strong modification signals were detected in reactions containing FicY from *Y. pseudotuberculosis* (Figure 3B top panel). In reactions containing Fic-2_E56G_ and ParE from *P. fluorescens*, weak modification signals were detected after exposure for over 13 h. Weak modification of SaGrlB which is the ParE equivalent in *S. aureus* was reproducibly detected when incubated with Sa1560 or its constitutively active mutant Sa1560_E107G_. In contrast, Fic proteins from neither *P. aeruginosa* nor *S. pneumoniae* detectably AMPylated their ParE counterparts (Figure 3B middle panel). These results indicate that, compared to GyrB, more Fic proteins (Fic-1, Fic-2_E56G_, FicY, and Sa1560_E107G_) AMPylate ParE or its counterpart GrlB in Gram-positive bacteria, suggesting that topoisomerase IV is the preferred target for the examined Fic proteins.

### Fic Proteins AMPylate ParE on a Tyrosine Residue Important for ATP Binding

**FIGURE 4:**
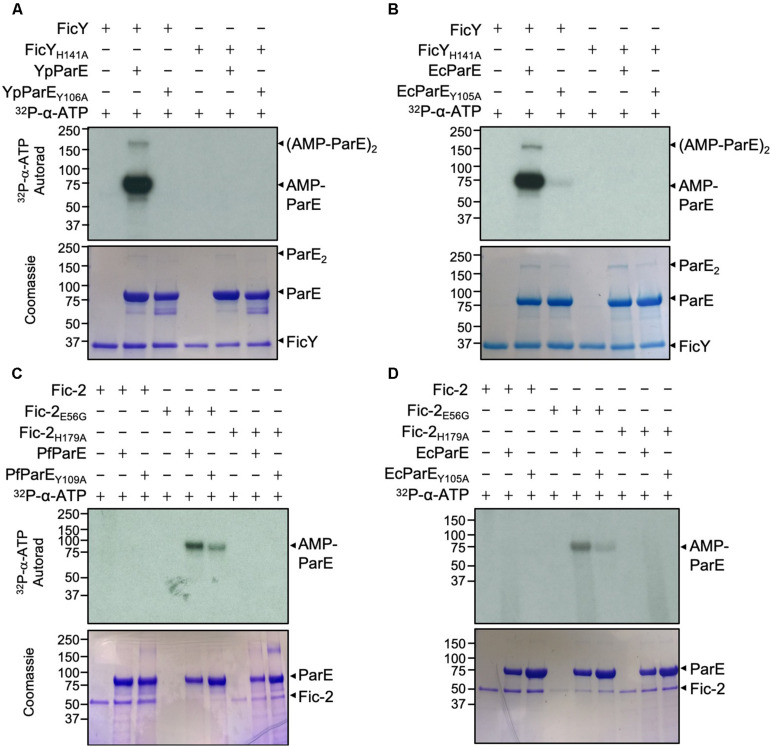
FicY and Fic-2 modified YpParE at Tyr^106^ and PfParE at Tyr^109^. **(A,B)** FicY from *Y. pseudotuberculosis* AMPylates YpParE at Tyr^106^ and EcParE at Tyr^105^. **(C,D)** Fic-2 of *P. fluorescens* modified PfParE at Tyr^109^ and EcParE at Tyr^105^. Signals were detected by 2.5 h **(A,C,D)** and 5 min **(B)** exposure. The predicted molecular mass of His_6_-SUMO tagged FicY, Fic-2, PfParE, EcParE, and YpParE is 37.44, 52.20, and 83.33, 84.04, and 83.79 kDa, respectively. Note that the Y106A or Y109A mutant of YpParE or PfParE can no longer be modified by FicY or Fic-2. The constitutively active form of Fic-2 (Fic-2_E56G_) significantly modified wild type PfParE and EcParE but not PfParE_Y109A_ and EcParE_Y105A_.

Given the high degree similarity between GyrB and ParE, we predicted that FicY modifies YpParE at Tyr^106^, a site that is equivalent to Tyr^111^ modified by Fic-1 in GyrB from *P. fluorescens*. To test this hypothesis, we created the YpParE_Y106A_ mutant. In reactions containing YpParE and ^32^P-a-ATP, robust modification was detected in reactions containing FicY, but not the FicY_H141A_ mutant in which the histidine residue within the core Fic motif was mutated into alanine. No modification signal was detected when YpParE_Y106A_ was used (Figure 4A), validating that Tyr106 of YpParE is the modification site. Interestingly, when EcParE_Y105A_ was used in the reaction, weak signals were still detected, suggesting that in addition to Tyr^106^, FicY modifies additional site(s) in EcParE at lower efficiencies (Figure 4B). Thus, Tyr^106^ is the major YpParE site modified by FicY.

When Fic-2_E56G_ was incubated with PfParE or EcParE in AMPylation reactions; weaker signals were detected in reactions containing Fic-2_E56G_ and PfParE_Y109A_ or Fic-2_E56G_ and EcParE_Y105A_. No signal was detected when Fic-2_H179A_ was used in the reactions (Figures 4C,D). These results suggest that more than one amino acid is AMPylated in PfParE by Fic-2_E56G_.

In reactions containing Sa1560 and SaGrlB or SaGrlB_Y109A_, Sa1560_E107G_ and SaGrlB or SaGrlB_Y109A_, weak signals of AMPylated SaGrlB and auto-modification signals were seen for Sa1560 from *S. aureus* strain USA300, which appeared to occur independently of its inhibitory domain (Supplementary Figure S3).

### AntY Inhibits the Activity of FicY

Sequence analysis revealed that an open reading frame downstream of *ficY* encodes a small protein that contains the -S_22_TAIET_27_- motif found in canonical Fic inhibitor elements (S/T)xxxE(G/N) (x, any amino acid) (Engel et al., 2012). This gene, designated *antY* (anti-*ficY*), is transcribed divergently from *ficY*. As expected, the inclusion of AntY in reactions containing YpParE and FicY led to complete inhibition of the modification, which is similar to the inhibition of Fic-1 activity by AntF (Supplementary Figure S4A) (Lu et al., 2016). Furthermore, the inhibition by AntY is dose-dependent, and the activity was completely inhibited when the molar ratio between FicY and AntY reached 1:3 (Supplementary Figure S4B). Thus, FicY, and AntY constitute a type II toxin and antitoxin module.

### Fic Proteins Affect DNA Topology *in vitro*

In bacteria, DNA gyrase is responsible for the introduction of negative supercoiling into relaxed DNA, and Topo IV catalyzes the relaxation of supercoiled DNA and decatenation of newly replicated DNA (Gellert et al., 1976; Kato et al., 1990). The observation that GyrB and ParE are targeted by Fic proteins prompted us to test how such modification affects their activity by examining their impact on the topological status of plasmid DNA.

**FIGURE 5:**
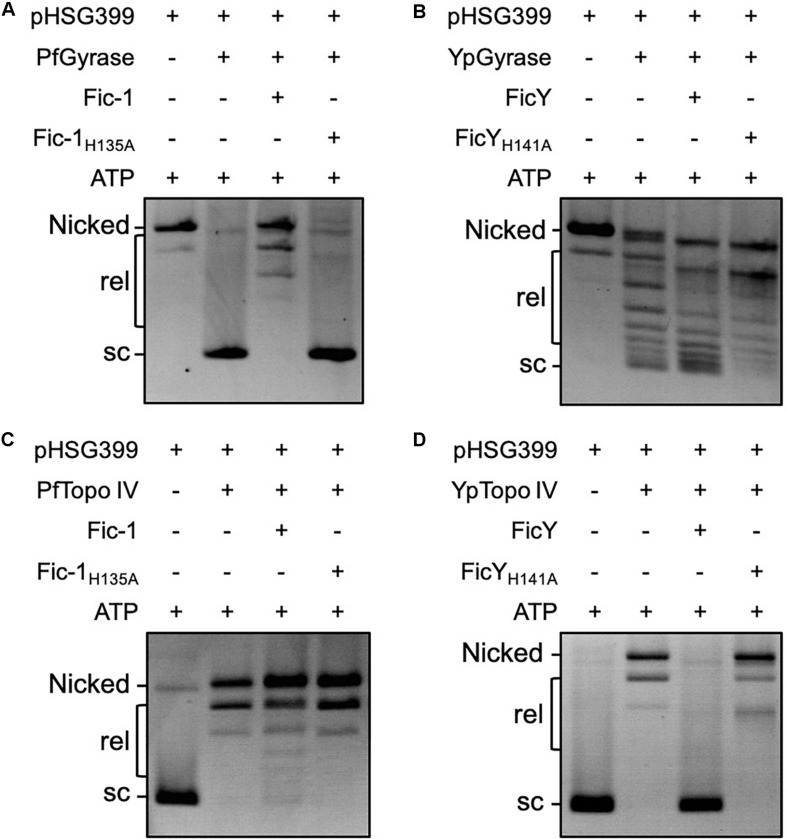
Fic proteins block the supercoiling activity of DNA gyrase and the relaxation activity of Topo IV. DNA gyrase (a mixture of the purified GyrA and GyrB) of either *P. fluorescens* **(A)** or *Y. pseudotuberculosis* **(B)** was pre-incubated with Fic-1 or FicY with 4 mM ATP at 30°C for 1 h. 0.3 μg relaxed plasmid DNA was added and reactions were allowed to proceed for 1 h. The reaction was stopped by 20 mM EDTA in sample buffer. Samples were separated by electrophoresis at 3 V/cm for 6–8 h. The activity of Topo IV of *P. fluorescens* **(C)** or *Y. pseudotuberculosis* **(D)** was similarly measured. Nicked: open circular DNA, rel: relaxed DNA, sc: supercoiled DNA.

DNA gyrase (GyrA and GyrB) from *P. fluorescens* (PfGyrase) introduced negative supercoiling into relaxed plasmid DNA. However, this activity became undetectable when equal amounts of Fic-1-treated PfGyrase were used. In line with its loss of catalytic activity, treatment with the Fic-1_H135A_ mutant did not affect the activity of PfGyrB (Figure 5A). Thus, AMPylation by Fic-1 abolishes DNA gyrase activity. Consistent with its inability to modify gyrase, pre-incubation of FicY with the DNA gyrase from *Y. pseudotuberculosis* (YpGyrase) did not affect its activity (Figure 5B). Furthermore, both Fic-1 and FicY had no effect on the relaxation activity of DNA gyrase in the absence of ATP (Supplementary Figure S5).

Inclusion of Fic-1 but not Fic-1_H135A_ in reactions containing Topo IV (ParC and ParE) proteins and supercoiled plasmid DNA led to detectable but not complete inhibition of the relaxation activity of the DNA topological enzymes from *P. fluorescens* (Figure 5C). In contrast, the activity of *Y. pseudotuberculosis* Topo IV can be completely inhibited by FicY after incubation for a similar duration and such inhibition required a functional Fic domain (Figure 5D).

### Inhibition of Topo IV by Fic Proteins Causes Cell Filamentation and Induces the SOS Response

**FIGURE 6:**
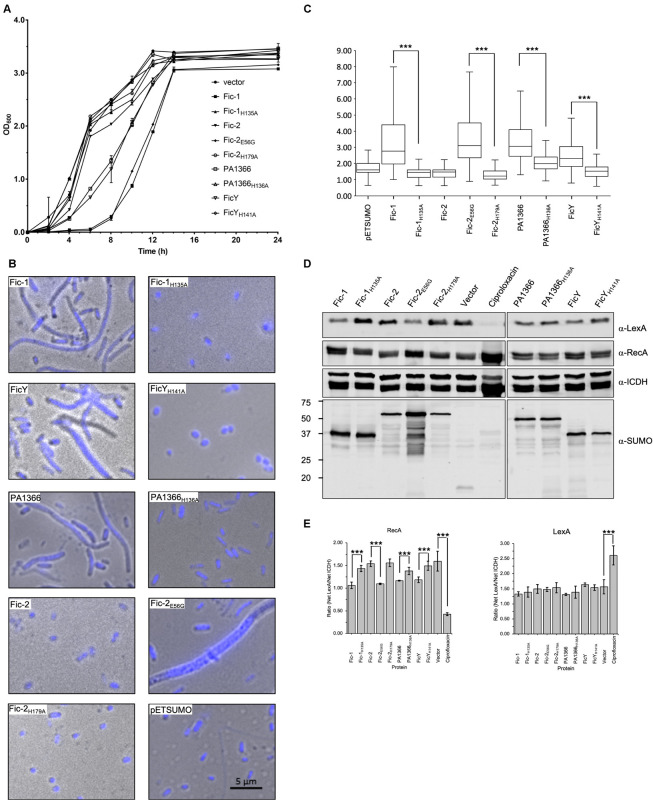
Fic proteins inhibit the growth of *E. coli*, cause cell filamentation and SOS induction. **(A)** Cells from plates were suspended in PBS and diluted into LB broth to identical densities. Growth was monitored by measuring OD_600_ at 2 h intervals. Note that the growth of cells from the vector expressing FicY, PA1366, Fic-2_E56G_, or Fic-1 was strongly inhibited. **(B)** Cells from *E. coli* transformants expressing SUMO-tagged each Fic protein or its mutant were grown for 16 h on LB agar, suspended in PBS, then fixed and stained with Hoechst. Note that expression of FicY, PA1366, Fic-2_E56G_, or Fic-1 caused cell filamentation **(C)** The length of 300 cells was measured from each sample, and their distribution was plotted. **(D,E)** Induction of the SOS response by Fic proteins or their mutant derivatives was performed as described previously (Lu et al., 2016). Cells expressing the indicated proteins were probed for LexA and RecA, respectively, with ICDH as a loading control **(D)**. The signal intensity of the bands from three independent experiments was measured by ImageJ and intensity ratios of relevant proteins were plotted **(E)**. Uncropped scans of images for all the blots were shown in Supplementary Figure S9. Source Data for Figure 6D have been provided as Supplementary Table S3. ICDH served as a loading control (third panel), expression levels of Fic proteins were determined by SUMO-specific antibody (last panel). Cells treated and untreated with 0.25 μg/mL ciprofloxacin were used as the positive control and negative control, respectively. The band intensities of LexA (First panel) and RecA (second panel) were measured by image J. The predicted molecular mass of LexA, RecA, ICDH, and His_6_-SUMO tagged Fic-1, Fic-2, PA1366, and FicY is 22.36, 37.97, 45.76, 37. 14, 52.20, 43.06, and 37.44 kDa, respectively. Note that the SOS pathway was induced in samples expressing Fic-2_E56G_ or Fic-1. All results were from three independent experiments, and represent data was shown. ***p < 0.01.

Inactive ParE is unable to relax positive supercoils in DNA, which will impact the segregation of newly replicated chromosomes, leading to the accumulation of DNA at the center of the cell, inhibition of cell growth and eventually the generation of filamentous cells (Bahng et al., 2000). To test whether Fic-mediated AMPylation of ParE affects the biological function of Topo IV, we expressed Fic proteins and their mutants in the *E. coli* strain BL21(DE3). Strains harboring plasmids expressing tagged *fic-1* or *fic-2*_E56G_ gene formed smaller colonies on LB plates. In contrast, cells expressing other Fic proteins formed colonies with sizes similar to those containing an empty vector (Supplementary Figure S6). Induction of the expression of proteins FicY, Fic-2_E56G_, PA1366, and Fic-1 by IPTG led to cell growth arrest in liquid cultures. No such inhibition was observed in strains carrying FicY_H141A_, PA1366_H136A_, Fic-2_H179A_, or Fic-1_H141A_, which grew indistinguishably to the control strain harboring the empty vector (Figure 6A).

We also examined the cell morphology by microscopic analysis and found that cells expressing FicY, Fic-2_E56G_, PA1366, or Fic-1 became filamentous. The typical *par* phenotype characterized by filamentous cells and un-segregated nucleoids was apparent in strains expressing Fic-2_E56G_. In contrast, no filamentous cell was found in strains expressing FicY_H141A_, Fic-2_H179A_, PA1366_H136A_, or Fic-1_H135A_ (Figure 6B). Cells from samples expressing active Fic proteins were significantly (*p* < 0.05) longer than those expressing their inactive mutants (Figure 6C and Supplementary Figure S7). We also examined the induction of the SOS response in these strains and found that the levels of RecA increased in cells expressing Fic-2_E56G_, Fic-1, PA1366, FicY which is similar to cells treated with the antibiotic ciprofloxacin (0.25 μg/mL). Consistent with other phenotypes, expression of Fic-2, Fic-2_H179A_, Fic-1_H135A_, PA1366_*H136A*_, or FicY_H141A_ did not induce the SOS response (Figures 6D,E). These results indicate that several Fic proteins target proteins involved in DNA replication and segregation, leading to cell filamentation and the induction of SOS response.

**FIGURE 7:**
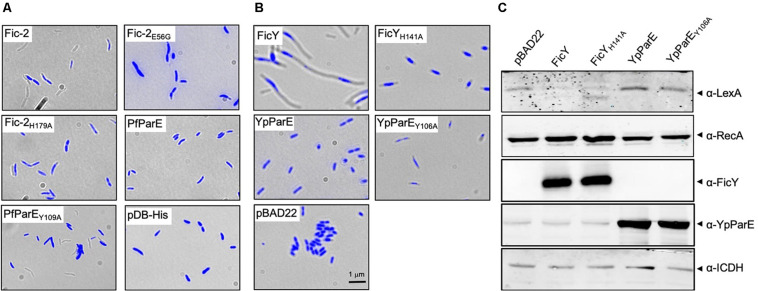
ParE-targeting Fic proteins caused compact and centrally located nucleoids in bacterial cells. **(A)** Each of the indicated genes was expressed from the arabinose inducible promoter in *P. fluorescens*. Cells grown to saturation in L. broth supplemented with 2% glucose were diluted 10-fold in ABM medium containing 0.2% arabinose for an additional 8 h. Cells washed with PBS were fixed using 4% paraformaldehyde and stained with Hoechst for imaging. **(B)** The indicated genes were expressed from the arabinose-inducible promoter and prepared for imaging as described above. In each case, the images acquired with a fluorescence microscope were pseudocolored with the IPLab software. **(C)** Cells grown as described in panel **(B)** were processed for SDS-PAGE and the proteins were probed with antibodies against LexA, RecA, FicY, and YpParE, respectively. The predicted molecular mass of LexA, RecA, ICDH, and His_6_-SUMO tagged FicY and YpParE is 22.36, 37.97, 45.76, 37.14, and 83.79 kDa, respectively. The metabolic enzyme ICDH was used as a loading control (bottom panel). Similar results were obtained from two independent experiments, and representative data was shown.

We also examined the effect of Fic-2 and FicY on *P. fluorescens* and *Y. pseudotuberculosis*, respectively. Although no typical *par* phenotype was observed, more cells unable to retain the Hoechst stain (anucleoid cells) were found in samples overexpressing Fic-2. A similar phenotype was found in cells overexpressing PfParE_Y109A_. No such phenotype was observed in cells harboring the vector or overexpressing PfParE (Figure 7A). In contrast, overexpression of FicY in *Y. pseudotuberculosis* triggered a canonical *par* phenotype. Shorter cell length and compact nucleoids were observed in cells expressing FicY_H141A_ and ParE_Y105A_, but not in cells expressing YpParE (Figure 7B). To examine whether inhibition of ParE induced the SOS response, we probed the protein levels of RecA and LexA in cells expressing FicY and found that RecA was induced, which was accompanied by a decrease in LexA. Such changes did not occur in cells overexpressing YpParE or YpParE_Y106A_ (Figure 7C). We also observed slight elongation of cells expressing FicY_H141A_, which may be caused by titration of endogenous AntY by this mutant. These results indicate that the modification of YpParE by FicY can lead to the development of a typical *par* phenotype and the induction of the SOS response.

## Discussion

DNA gyrase and topoisomerase IV regulate underwound or overwound DNA during chromosome replication, segregation, and transcription, and are targeted by many antibacterial agents as well as toxins encoded by genes on bacterial chromosomes or plasmids. Antibiotics targeting these enzymes include well-characterized quinolones and coumarins such as nalidixic acid, ciprofloxacin, novobiocin and clorobiocin, which function by competing with ATP for the binding site on the B subunit (Mayer and Janin, 2014; Hooper and Jacoby, 2016). Plasmid-born toxins such as ParE_RK2_, CcdB_F_ and microcins inhibit the supercoiling activity of DNA gyrase by either targeting the subunit(s) of gyrase or the DNA-gyrase cleavage complex (Dao-Thi et al., 2005; Pierrat and Maxwell, 2005; Deghorain et al., 2013; Metelev et al., 2013; Bush et al., 2015). A recently identified antibiotics and phytotoxin albicidin from *Xanthomonas albilineans* interferes with the catalytic DNA cleavage-religation cycle of the GyrA subunit of DNA gyrase and traps it in a conformation that differs from that targeted by quinolones (Cociancich et al., 2015).

Here, we found that Fic-1 from *P. fluorescens* inhibits DNA replication by targeting both GyrB and ParE at a highly conserved Tyr residue. This property is similar to VbhT, a Fic protein from *Bartonalla schoenbuchensis* that also AMPylates both GyrB and ParE (Harms et al., 2015). In our assays, we consistently observed that the activity of Fic-1 toward GyrB appears more robust than that toward ParE. In line with the observation that targeting GyrB by Fic-1 abolished its ATPase activity, the supercoiling activity of the gyrase is completely abolished upon AMPylation induced by Fic-1. In contrast, AMPylation of ParE has little effects on the supercoiling relaxation activity.

Differing from Fic-1 from *P. fluorescens*, FicY of *Y. pseudotuberculosis* prefers ParE (Figure 3B). AMPylation of ParE by FicY blocked the relaxation ability of Topo IV, leading to the development of a typical *par* phenotype characterized by cell filamentation and unsegregated nucleoids (Figure 7B). The relationship between FicY and AntY and organization of their genes suggest that these two proteins form a canonical type II toxin-antitoxin system. The *ficY* and *antY* genes transcribe convergently, which differs from *fic-1* and *antF* that appears to be controlled by the same promoter (Supplementary Figure S2) (Lu et al., 2016). Apparently, the expression of *ficY* and *antY* is controlled by independent promoters, which may allow more specific regulation.

Some Fic proteins such as dFic from *Drosophila*, HYPE, and EfFIC from *Enterococcus faecalis* harbor an intramolecular inhibitory motif and their activity is influenced by the oligomeric state of the enzyme and the presence of certain metal ions (Casey et al., 2017; Preissler et al., 2017; Perera et al., 2019; Veyron et al., 2019). Similar to these proteins, Fic-2 harbors an inhibitory motif in its amino terminal portion capable of blocking its AMPylation activity. Mutations that abolished the predicted inhibitory motif allowed detectable modification of ParE by Fic-2 at levels considerably lower than those of Fic-1 or FicY. Interestingly, albeit both Fic-2_E56G_ and Fic-1 AMPylate ParE, these two proteins appear to differently impact cellular processes of *E. coli* and *P. fluorescens*. In *E. coli*, ectopic expression of Fic-1 causes filamentation (Lu et al., 2016), whereas Fic-2_E56G_ induces a typical *par* phenotype. In *P. fluorescens*, expression of Fic-2 led to the formation of cells without nucleoids, suggestive of inhibition of DNA separation (Figure 7A). Of note is that the carboxyl-terminal portion of Fic-2 is predicted to contain a helix-turn-helix motif often involved in DNA binding, yet the role of this putative motif in bacterial physiology, if any, awaits further investigation. A recent study found that the AMPylation activity of Fic protein from *Clostridium difficile* occurs independently of its predicted inhibitory domain (Dedic et al., 2016). Interestingly, self-modification of Sa1560 appeared to occur in reactions containing wild type protein, suggesting that the AMPylation activity of Sa1560 is independent of its inhibitory motif.

Only a fraction of Fic proteins tested in this study AMPylate the DNA gyrase or topoisomerase IV. Those that display undetectable activity against these two enzymes involved in DNA topology may have different cellular targets or enzymatic activities distinct from AMPylators. In our auto-AMPylation reactions, only Fic-1, Fic-2, FicY, and Sa1560 showed auto-AMPylation activity. Introduction of mutations that abolished the predicted intramolecular inhibitory motifs of class II (Fic-2 and Fic-3 from *P. fluorescens*; PA0547 from *P. aeruginosa*) and class III (Sp0496 from *S. pneumoniae*) did not lead to proteins with detectable activity (Supplementary Figure S8). In addition, overexpression of these Fic proteins or their presumably constitutively active mutants in *E. coli* did not affect cell division (Supplementary Figure S7). Theses Fic proteins may target proteins other than GyrB and ParE, and the lack of auto-AMPylation activity suggests that they have biochemical activities distinct from AMPylators. Alternatively, our experimental conditions may not be suitable for the detection of their activity.

**FIGURE 8:**
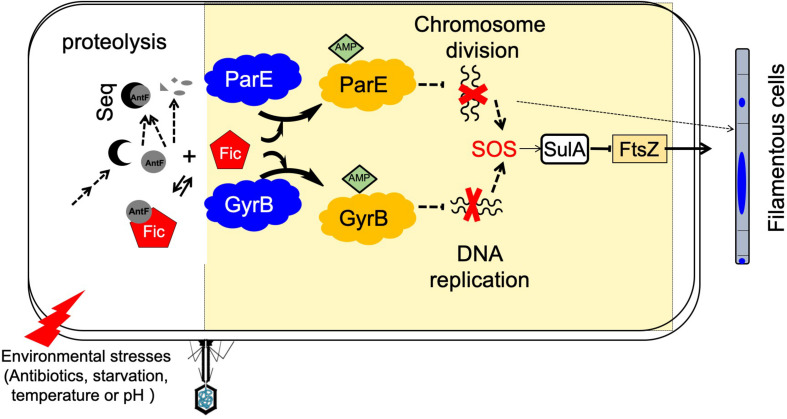
A model for Fic-mediated induction of bacterial cell filamentation and its regulation. Fic-1 and AntF form a dynamic complex under normal conditions in the cell. Potential signals from the environment such as phage infection, starvation, antibiotics, changes in temperature or pH, activate a signaling cascade that leads to the production of a sequestering protein (Seq) that competes for AntF or the activation of a protease that degrades AntF. Freed or activated Fic-1 then inactivates ParE or GyrB by AMPylation, following by inhibition of DNA replication and separation, and leading to initiation of the SOS response, which includes the induction of SulA and the formation of filamentous cells. Alternatively, inactivation of ParE or GyrB by AMPylation may induce cell filamentation through an SOS response–independent pathway (dashed arrows).

In line with their inhibition of the enzymatic activity of GyrB and ParE, overexpression of the Fic proteins robustly induced the SOS response (Figure 8). Blockage of DNA replication by Fic proteins likely leads to the accumulation of ssDNA, which is the direct effects of physical and chemical assaults mediated by UV radiation or antibiotics such as mitomycin C (Dörr et al., 2009). We propose that one or more yet unidentified cues induce the expression of the Fic genes or tilts the balance between a Fic protein and its inhibitor in a direction that favors Fic activity, leading to temporal inhibition of DNA replication, which gives the affected cells a better chance to survive.

AMPylation induced by Fic proteins or other AMPylators is reversible by specific enzymes (Tan and Luo, 2011). Recent interesting studies reveal that the human Fic protein HYPE displays a deAMPylase activity in response to fluctuations in the unfolded protein load (Casey et al., 2017; Preissler et al., 2017; Perera et al., 2019; Veyron et al., 2019). Whether and how the activity of Fic proteins from other organisms is regulated awaits further investigation.

## Data Availability Statement

The original contributions presented in the study are included in the article/Supplementary Material, further inquiries can be directed to the corresponding authors.

## Author Contributions

C-HL, Z-QL, and L-QZ designed the research and analyzed the data. C-HL, AM, EN, and F-RC performed the research. C-HL, AM, L-QZ, and Z-QL wrote the manuscript. All authors contributed to the article and approved the submitted version.

## Conflict of Interest

The authors declare that the research was conducted in the absence of any commercial or financial relationships that could be construed as a potential conflict of interest.
