# Supplementary material for: Fic Proteins Inhibit the Activity of Topoisomerase IV by AMPylation in Diverse Bacteria
Source: Front Microbiol. 2020 Aug 26;11:2084. doi: 10.3389/fmicb.2020.02084 (PMC7479194; doi:10.3389/fmicb.2020.02084)
Supplement: Supplementary file 1 [file Data_Sheet_1.docx]

Supplementary Material

**Fic proteins inhibit the activity of topoisomerase IV by AMPylation in diverse bacteria**

Can-Hua Lu^1,2,3^, Alix McCloskey^3^, Fu-Rong Chen^2^, Ernesto S. Nakayasu^4^, Li-Qun Zhang^2^ and Zhao-Qing Luo ^3^

^1^Yunnan Academy of Tobacco Agriculture Science, Kunming, China

^2^Department of Plant Pathology and MOA Key Lab of Pest Monitoring and Green Management, College of Plant Protection, China Agricultural University, Beijing, China

^3^Department of Biological Sciences, Purdue University, West Lafayette, IN, USA

^4^Biological Science Division, Pacific Northwest National Laboratory, Richland, WA, USA

### **Supplementary Figures**

**Supplementary Figure 1. Sequence alignment of AMPylation sites with flanking sequences of GyrB and ParE, the Fic motifs and the inhibitory domains from several taxonomically diverse bacteria**. (**A-C**) The subunit B of DNA gyrase and Topo IV ParE from the indicated bacteria identified by PSI-BLAST searches were aligned by the Clustal W 2.1 program. (**D-E**) Fic domains and their putative inhibitory motifs from indicated bacteria were aligned. Identical residues are highlighted in light yellow background. Residues critical for catalysis or inhibition are marked in black background. The proteins included are as follows: Fic-1 (AMR99724), Fic-2 (AMR99725), Fic-3 (AMR99726), PA1366 (NP_250057), PA0574 (NP_249265), EcFic (NP_417820), FicY (ACA66911), MT3743 (AAK48102), SA1560 (ABD21967), SPD_0496 (ABJ54309), AntF (AMR99723), YhfG (NP_417821), AntY (ACA66912), N297_1407 (WP_003102002), MT_RS19090 (WP_003419627), PfGyrB (AMR99727), PaGyrB (WP_003097268), EcGyrB (AMC96591), YpGyrB (AJJ59491), MtGyrB (AAK44228), SaGyrB (WP_000255586), SpGyrB (WP_000134039), PfParE (AUM67986), PaParE (NP_253654), EcParE (NP_417502), YpParE (WP_002212181), SaGrlB (AHJ07085), SpGrlB (WP_000037270).

**Supplementary Figure 2. Physical maps of Fic genes and their putative inhibitory genes.** Ten fic proteins were obtained from *P. fluorescens* 2P24, *P. aeruginosa* PAO1, *E. coli* DH5α, *Y. pseudotuberculosis* YPIII, *M. tuberculosis* CDC1551, *S. aureus* USA300 and *S. pneumoniae* D39. Fic motifs are highlighted in red, and their putative inhibition domains are shown in black. Fic genes were divided into three classes according to the position of the inhibitory motif along the polypeptide chain(Engel et al., 2012). The blowups indicate the sequences of the predicted Fic domain and its inhibitory motif.

**Supplementary Figure 3. The Fic protein from *S. aureus* strain USA300 AMPylates SaGrlB**. Three micrograms of Fic protein were incubated with 10 µg of SaGrlB or its mutant proteins for 30 min at 35°C. The signals of ^32^P-α-SaGrlB and total proteins were detected by autoradiography for 2.5 h (upper panel) and Coomassie blue staining (lower panel). The molecular mass of His_6_-SUMO-tagged Sa1560 and SaGrlB is 61.48 and 88.16 kDa, respectively. Note that strong auto-AMPylation signals were observed in lanes containing only Sa1560 or Sa1560_E107G_. Both Sa1560 and Sa1560_E107G_ AMPylate wide type and the Y109A mutant of SaGrlB. The physiological significance of the weak and modification of SaGrlB by Sa1560 is unclear.

**Supplementary Figure 4. The AMPylation of ParE by Fic-1 and FicY is inhibited by their cognate antitoxins**. **(A)** Three micrograms of the putative antitoxin of FicY (AntY) or AntF were incubated with 3 µg FicY or 1.5 µg Fic-1 for 30 min, followed by the addition of 10 µg of ParE for another 30 min. The samples were separated and AMPylation was detected as described above. Both AntY and AntF significantly inhibit the AMPylation activities of FicY and Fic-1, respectively, *in vitro*. **(B)** Dose-dependent inhibition of FicY toxin by AntY antitoxin. The indicated amounts of His_6_-SUMO-AntY were added to a series of identical reactions each containing 3 µg FicY. After 30 min of incubation, equal amounts of a mixture containing YpParE and ^32^P-α-ATP were added, and the reactions were allowed to proceed for 30 min at 35°C. ^32^P-α-YpParE and total proteins were detected by autoradiography (upper panel) and Coomassie blue staining (lower panel). The predicted molecular mass of Fic-1, and His_6_-SUMO tagged FicY, AntF and AntY is 22.34, 37.44, 20.18 and 19.45 kDa, respectively.

**Supplementary Figure 5. Fic-1 and FicY have no effect on the relaxation activity of DNA gyrase**. Relaxation of negative supercoils by DNA gyrase in an ATP-independent reaction was assayed in a reaction mixture (40 µl) containing 50 mM Tris-HCl, pH 7.9 at 25°C, 50 mM KOAc, 10 mM MgCl_2_, 0.1 mg/ml BSA. When needed, five micrograms of Fic-1 or FicY were added. The reaction was started by adding 0.3 µg negatively supercoiled pHSG399 DNA, incubated for 1 h at 30°C, and terminated by EDTA (final concentration of 20 mM). The reactions were analyzed by electrophoresis in 1.2% agarose gels at 45 V for 4 h. DNA agarose gels were stained with 1 µg/ml ethidium bromide for 20 min and images were acquired. Nicked: open circular DNA, rel: relaxed DNA, sc: supercoiled DNA.


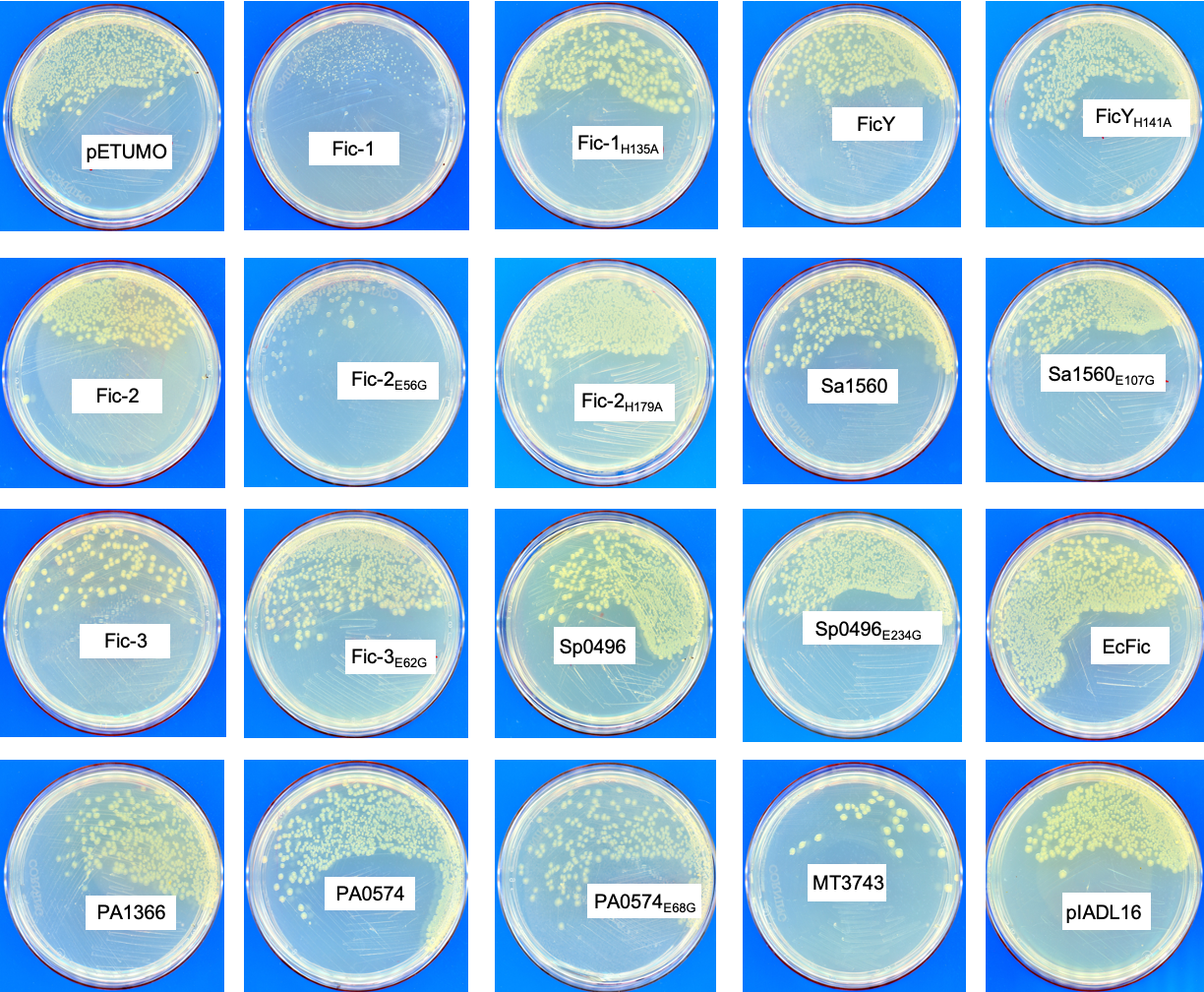


**Supplementary Figure 6. The growth of *E. coli* cells transformed with plasmids carrying Fic proteins or their mutants.** Chemical competent cells of the *E. coli* strain BL21(DE3) were transformed with derivatives of pET-SUMO harboring the indicated Fic genes. The empty vector pETSUMO was included as a control. Note that *MT3743* from *M. tuberculosis* CDC1551 was cloned in pIADL16 vector (McCafferty et al., 1997). The images were acquired after transformed cells were incubated at 37℃ for 16 h.

**Supplementary Figure 7. Length of the cell expressing Fic genes tested in this study**. Cells from *E. coli* transformants expressing SUMO-tagged Fic proteins or their mutants grown for 16 h on LB agar suspended in PBS were fixed and stained with Hoechst. Samples were inspected with an AxioObeserver Z1 fluorescence microscope, and images were acquired using a charge-coupled device camera with identical digital imaging parameters. The images and cell lengths were processed and measured using the ZEN pro 2011 software package (ZEISS). For each sample, the data were from 300 cells. SAS University Edition online software was used for Duncan grouping. Means covered by the same bar are not significantly different, *p*<0.05.


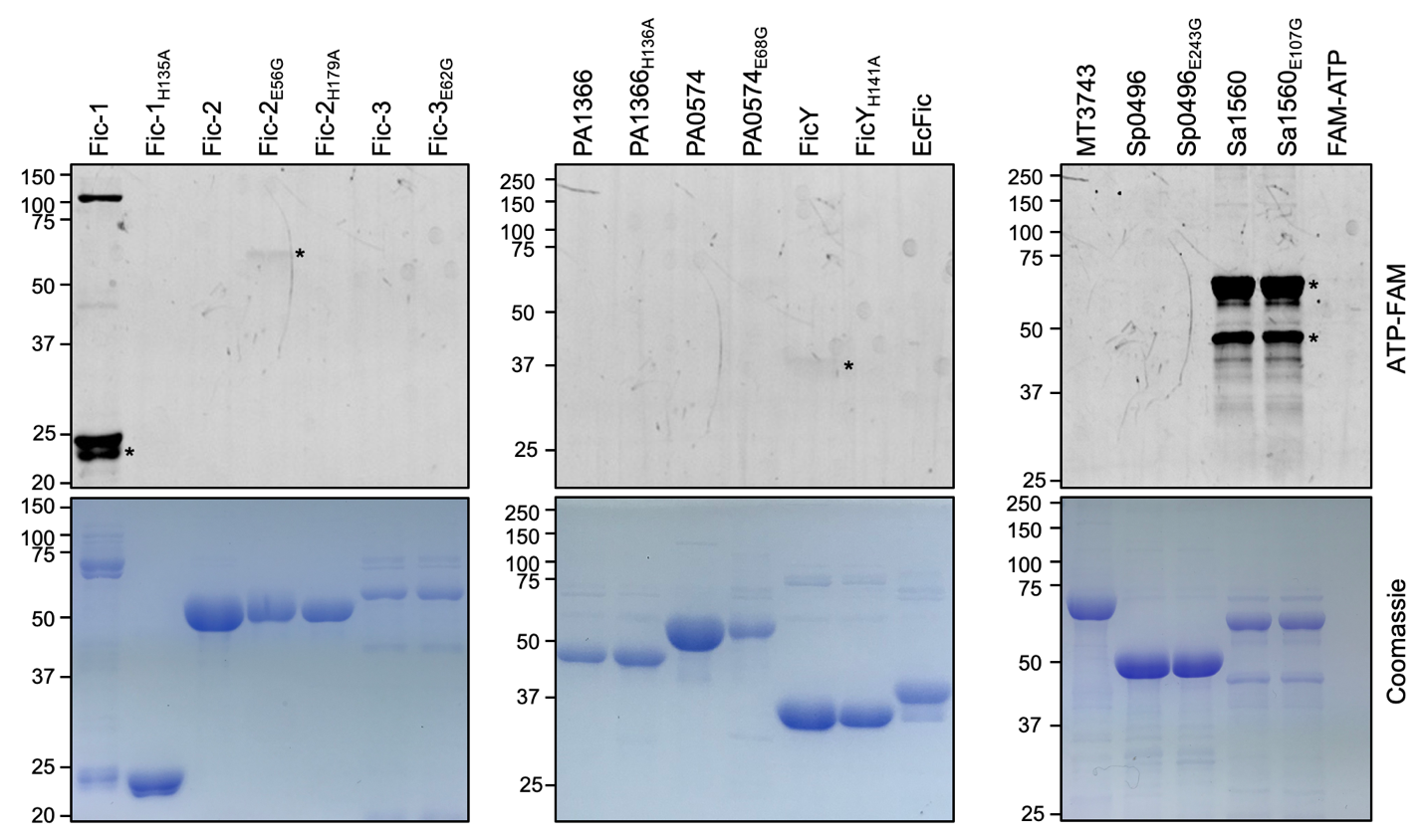


**Supplementary Figure 8. Auto-AMPylation of the Fic proteins analyzed in this study.** Ten micrograms of Fic proteins were incubated with 5 µM ATP-FAM at 35℃ for 1 h. After reactions were terminated with the Laemmli buffer, samples were boiled and separated on 12% SDS-PAGE gels. Fluorescence signals were determined by iBright FL1500 Imaging System, and protein levels were detected by Coomassie brilliant blue staining. Note that fluorescence signals (labeled with asterisks) were obtained from the lanes containing Fic-1, Fic-2_E56G_, FicY, Sa1560 or Sa1560_E107G_.

**Supplementary Figure 9. The effects of Fic proteins on the expression of RecA and LexA.** Total proteins from cells expressing the indicated Fic proteins were separated by SDS-PAGE and immunoblotted with antibodies against LexA, RecA, ICDH or SUMO. Proteins were detected with the fluorescence-labeled secondary antibody IRDye 700. The signals were detected with the LI-COR Odyssey Imaging system (Li-COR Biosciences). Cells treated and untreated with 0.25 µg/mL ciprofloxacin were used as positive control and negative control, respectively. The predicted molecular mass of LexA, RecA, ICDH, and His_6_-SUMO-tagged Fic-1, Fic-2, PA1366 and FicY is 22.36, 37.97, 45.76, 37. 14, 52.20, 43.06 and 37.44 kDa, respectively. Note that the SOS pathway was induced in samples expressing FicY, PA1366, Fic-2_E56G_ or Fic-1. Similar results were obtained from three independent experiments, and the results from one representative experiment were shown.

### **Supplementary Tables**

**Supplementary Table 1. Strains, genomic DNAs and plasmids used in this study**

| Name | Relevant characteristics | References or sources |
| --- | --- | --- |
| Strains |  |  |
| *Escherichia coli* DH5α | F-, *φ80dlacZΔM15*, *Δ*(*lacZYA-argF*)*U169*, *deoR*, *recA1*, *endA1*, *hsdR17*(*rk-*, *mk+*), *phoA*, *supE44*, *λ-*, *thi-1*, *gyrA96, relA1.* | (Hanahan, 1983) |
| *E. coli* BTH101 | Str^R^; F-, *cya-99*, *araD139, galE15, galK16, rpsL1*, *hsdR2, mcrA1, mcrB1.* | (Karimova et al., 1998) |
| *E. coli* BL21(DE3) | F-, *ompT*, *hsdS*(*rBB-mB-*), *gal*, *dcm*(*DE3*)*.* | Novagen |
| *Pseudomonas fluorescens* 2P24 | Amp^R^; wide type | (Wei and Zhang, 2006) |
| *P. aeruginosa* PAO1 | Wide type strain | Our collection |
| *Yersinia pseudotuberculosis* YPIII | Wide type strain | Our collection |
| Genomic DNAs |  |  |
| *Streptococcus pneumoniae* D39 | Genomic DNA | Dr. Xuming Deng (Jilin University) |
| *Staphylococcus aureus* USA300 | Genomic DNA | Dr. Xuming Deng (Jilin University) |
| *Mycobacterium tuberculosis* CDC1551 | Genomic DNA | Dr. David Russell (Cornell University) |
| Vectors |  |  |
| pET22b (+) | Amp^R^; expression vector | Novagen |
| pET-SUMO | Kan^R^; expression vector | Champion |
| pGEX-6P1 | Amp^R^; expression of GST-fusion protein | Our collection |
| pIADL16 | Amp^R^; expression of His_6_-MBP-fusion protein | (McCafferty et al., 1997) |
| pHSG399 | Cm^R^; ColE1 origin | TaKaRa |
| pBAD22 | Amp^R^; tight regulation of protein expression by vector containing the arabinose P_BAD_ promoter | (Guzman et al., 1995) |
| pCL008 | Kan^R^; pBBR1MCS-2 containing the arabinose P_BAD_ promoter from pBAD22 vector | (Lu et al., 2016) |

**Supplementary Table 2. Primers used in this study**

| Primer | Sequence | Purpose |
| --- | --- | --- |
| pET-SUMO-F | TGAAGATTTGGACATGGAGG | For sequencing |
| pET-SUMO-R | CAGCAGCCAACTCAGCTTCC |  |
| pDB-His-F | CAAAGCGGGACCAAAGCCATGAC | For sequencing |
| pDB-His-R | CAATTTCCATTCGCCATTCAGG |  |
| EcParE-*Xba I*-F | ACTCTAGAGACGCAAACTTATAACGCTGATG | 5’ primer for cloning *parE* of *E. coli* onto pUT18C |
| EcParE-*EcoR I*-R | ATGAATTCTTAAACCTCAATCTCCGCCATG | 3’ primer for cloning *parE* of *E. coli* onto pUT18C |
| PfFic-1-*Xba I*-F | ATTCTAGAGCCTGACAAATATGGGGTCG | 5’ primer for cloning *fic-1* of *P. fluorescens* onto pKT25 or pUT18C |
| PfFic-1-*Kpn I*-R | ATTGGTACCTCAAGCTTGAATCGCCTGCCC | 3’ primer for cloning *fic-1* of *P. fluorescens* onto pKT25 or pUT18C |
| PfAntF-*BamH I*-F | ATGGATCCCGGCAATGTCAGCCTTGAAACC | 5’ primer for cloning *antF* of *P. fluorescens* onto pKT25 or pUT18C |
| PfAntF-*EcoR I*-R | ATGAATTCAGGTTCGGGTCTGGGTGAAG | 3’ primer for cloning *antF* of *P. fluorescens* onto pKT25 or pUT18C |
| PfFic-1-*Nde I*-F | ATTCATATGCCTGACAAATATGGGGTCG | 5’ primer for cloning *fic-1* of *P. fluorescens* onto pET-22b (+) |
| PfFic-1-*Sal I*-R | ATTGTCGACAGCTTGAATCGCCTGCCCGA | 3’ primer for cloning *fic-1* of *P. fluorescens* onto pET-22b (+) |
| PfFic-2-*BamH I*-F | ATTGGATCCATGAGTCGTTATACGCCAC | 5’ primer for cloning *fic-2* of *P. fluorescens* onto pET-SUMO |
| PfFic-2-*Sal I*-R | ATTGTCGACTCAGCGAGGGCGCACCGAT | 3’ primer for cloning *fic-2* of *P. fluorescens* onto pET-SUMO |
| PfFic-3-*Bgl II*-F | ATTAGATCTATGATCACCTCGAAAACGTAC | 5’ primer for cloning *fic-3* of *P. fluorescens* onto pET-SUMO |
| PfFic-3-*Sal I*-R | ATTGTCGACTTAGCCCATTTGCAAGTGCAG | 3’ primer for cloning *fic-3* of *P. fluorescens* onto pET-SUMO |
| PfFic-3-*Hind III*-F | ATTAAGCTTCTGCCGCCAGGTCGCCC | 5’ primer for cloning *fic-3* of *P. fluorescens* onto pHSG399 |
| PfFic-3-*Xba I* | ATTCTAGACAGGTTCGTTCTGTTCAGTCG | 3’ primer for cloning *fic-3* of *P. fluorescens* onto pHSG399 |
| PfAntF-*BamH I*-F | ATTGGATCCATGGGCAATGTCAGCCTTGA | 5’ primer for cloning *antF* of *P. fluorescens* onto pET-SUMO |
| PfAntF-*Sal I*-R | ATTGTCGACTCAGGTTCGGGTCTGGGTG | 3’ primer for cloning *antF* of *P. fluorescens* onto pET-SUMO |
| EcFic-*BamH* I-F | ATTGGATCCATGAGCGATAAATTCGGCG | 5’ primer for cloning *fic* from *E. coli* onto pET-SUMO |
| EcFic-*Sal* I-R | ATTGTCGACTTACTCAGATTCCCCGGCTTC | 3’ primer for cloning *fic* from *E. coli* onto pET-SUMO |
| Sa1560-F | ATTGGATCCATGGGTTACAGAACTTTAAAAAG | 5’ primer for cloning *Sa1560* from *S. aureus* onto pET-SUMO |
| Sa1560-*Sal I*-R | ATTGTCGACTTATTTTATAATCGAATTTAGAAATTCATCAC | 3’ primer for cloning *Sa1560* from *S. aureus* onto pET-SUMO |
| Sp0496-*BamH I*-F | ATTGGATCCATGCAACCAACTTACAACATTGAC | 5’ primer for cloning *Sp0496* from *S. pneumoniae* onto pET-SUMO |
| Sp0496-*Sal I*-R | ATTGTCGACTTATGAAAAATCGAGGTCTAAATC | 3’ primer for cloning *Sp0496* from *S. pneumoniae* onto pET-SUMO |
| PA1366-*BamH I*-F | ATTGGATCCATGAGTTTCGATCCCTTTGG | 5’ primer for cloning *Pa1366* from *P. aeruginosa* onto pET-SUMO |
| PA1366-*Sal I*-R | ATTGTCGACTTAGGGTTCGGAGTCGGGTG | 3’ primer for cloning *Pa1366* from *P. aeruginosa* onto pET-SUMO |
| PA0574-*BamH I*-F | ATTGGATCCATGAACGACCCACTCTGG | 5’ primer for cloning *Pa0574* from *P. aeruginosa* onto pET-SUMO |
| PA0574-*Sal I*-R | ATTGTCGACTCAGCGTGCTATCTGGTAACG | 3’ primer for cloning *Pa0574* from *P. aeruginosa* onto pET-SUMO |
| FicY-*BamH I*-F | ATTGGATCCATGACAAAAAAAACAGTACACAG | 5’ primer for cloning *YPK_0608* from *Y. pseudotuberculosis* onto pET-SUMO |
| FicY-*Sal I*-R | ATTGTCGACCTAGCGGGCGAGGCCAAC | 3’ primer for cloning *YPK_0608* from *Y. pseudotuberculosis* onto pET-SUMO |
| EcGyrA-*BamH I*-F | ATTGGATCCATGAGCGACCTTGCGAGAG | 5’ primer for cloning *gyrA* of *E. coli* onto pET-SUMO |
| EcGyrA-*Sal I*-R | ATTGTCGACTTATTCTTCTTCTGGCTCGTC | 3’ primer for cloning *gyrA* of *E. coli* onto pET-SUMO |
| EcGyrB-*BamH I*-F | ATTGGATCCATGTCGAATTCTTATGACTCC | 5’ primer for cloning *gyrB* of *E. coli* onto pET-SUMO |
| EcGyrB-*Xho I*-R | ATTCTCGAGTTAAATATCGATATTCGCCGC | 3’ primer for cloning *gyrB* of *E. coli* onto pET-SUMO |
| EcParE-*Bgl II*-F | ATTAGATCTATGACGCAAACTTATAACGCT | 5’ primer for cloning *parE* of *E. coli* onto pET-SUMO |
| EcParE-*Sal I*-R | ATTGTCGACTTAAACCTCAATCTCCGCCATG | 3’ primer for cloning *parE* of *E. coli* onto pET-SUMO |
| PfGyrA-*Bgl II*-F | ATTAGATCTATGGGCGAACTGGCCAAAGA | 5’ primer for cloning *gyrA* of *P. fluorescens* onto pET-SUMO |
| PfGyrA-*Sal I*-R | ATTGTCGACTTAGTCCTGCGGTTCTTCTTC | 3’ primer for cloning *gyrA* of *P. fluorescens* onto pET-SUMO |
| YpGyrA-*BamH I*-F | ATTGGATCCATGAGTGACTTGACTCATGAC | 5’ primer for cloning *gyrA* of *Y. pseudotuberculosis* onto pET-SUMO |
| YpGyrA-*Sal I*-R | ATTGTCGACTTACTCTTCGCTCTCGGTGG | 3’ primer for cloning *gyrA* of *Y. pseudotuberculosis* onto pET-SUMO |
| MtGyrB-*Bgl II*-F | ATTAGATCTATGCACGCAACCCCTGAGG | 5’ primer for cloning *gyrB* of *M. tuberculosis* onto pET-SUMO |
| MtGyrB-*Xho I*-R | ATTCTCGAGTTAGACATCCAGGAACCGAAC | 3’ primer for cloning *gyrB* of *M. tuberculosis* onto pET-SUMO |
| PaGyrB-*Sac I* F | ATTGAGCTCATGAGCGAGAACAACACGTACG | 5’ primer for cloning *gyrB* of *P. aeruginosa* onto pET-SUMO |
| PaGyrB-*Xho I* R | ATCCTCGAGTCACACGTCCAGGTTCGACACCGC | 3’ primer for cloning *gyrB* of *P. aeruginosa* onto pET-SUMO |
| PfGyrB-U-*BamH I*-F | ATTGGATCCTTGAGCGAAGAAAATACGTACG | 5’ primer for cloning the N-terminus of *gyrB* onto pET-SUMO |
| PfGyrB-U-*Age I*-R | GCATCGTCACCGGTGGTGG | 3’ primer for cloning the N-terminus of *gyrB* onto pET-SUMO |
| PfGyrB-D-*Age I*-F | CCACCACCGGTGACGATGC | 5’ primer for cloning the C-terminus of *gyrB* onto pET-SUMO |
| PfGyrB-D-*Xho I*-R | ATACTCGAGTCAGAAATCCAGGTTGGACAC | 3’ primer for cloning the C-terminus of *gyrB* onto pET-SUMO |
| PfParE-U-*Bgl II*-F | ATTAGATCTATGGCCACTCCCAGCGCTAGC | 5’ primer for cloning the N-terminus of *parE* onto pET-SUMO |
| PfParE-U-*Age I*-R | CAGGGCCAAACCGGTTTCC | 3’ primer for cloning the N-terminus of *parE* onto pET-SUMO |
| PfParE-D-*Age I*-F | GGAAACCGGTTTGGCCCTG | 5’ primer for cloning the C-terminus of *parE* onto pET-SUMO |
| PfParE-D-*Xho I*-R | ATTCTCGAGTCAGGCCAGCACCTCAGC | 3’ primer for cloning the C-terminus of *parE* onto pET-SUMO |
| SaGyrB-*BamH I*-F | ATTGGATCCATGGTGACTGCATTGTCAGATG | 5’ primer for cloning *gyrB* of *S. aureus* onto pET-SUMO |
| SaGyrB-*Sal I*-R | ATTGTCGACTTAGAAGTCTAAGTTTGCATAAACTGC | 3’ primer for cloning *gyrB* of *S. aureus* onto pET-SUMO |
| SpGyrB-U-*BamH I*-F | ATTGGATCCATGACAGAAGAAATCAAAAATCTGCAG | 5’ primer for cloning the N-terminal *gyrB* of *S. pneumoniae* onto pET-SUMO |
| SpGyrB-U-*BstE II*-R | TTTCATGGTAACCCGTTGTG | 3’ primer for cloning the N-terminal *gyrB* of *S. pneumoniae* onto pET-SUMO |
| SpGyrB-D-*BstE II*-F | GTACACAACGGGTTACCATG | 5’ primer for cloning the C-terminal *gyrB* of *S. pneumoniae* onto pET-SUMO |
| SpGyrB-D-*Sal I*-R | ATTGTCGACTTAGACATCAAGTGTACTATAGACAG | 3’ primer for cloning the C-terminal *gyrB* of *S. pneumoniae* onto pET-SUMO |
| PfParC-*BamH I*-F | ATTGGATCCATGAGCGACATTCTTGCAGAC | 5’ primer for cloning *parC* of *P. fluorescens* onto pET-SUMO |
| PfParC-*Xho I*-R | ATACTCGAGCTAATTGAGGTTTTCGACGAG | 3’ primer for cloning *parC* of *P. fluorescens* onto pET-SUMO |
| YpParC-*BamH I*-F | ATTGGATCCATGAGTGACTTGACTCATGACGGTG | 5’ primer for cloning *parC* of *Y. pseudotuberculosis* onto pET-SUMO |
| YpParC-*Sal I*-R | ATTGTCGACTTACTCTTCGCTCTCGGTGG | 3’ primer for cloning *parC* of *Y. pseudotuberculosis* onto pET-SUMO |
| EcParC-*BamH I*-F | ATTGGATCCATGAGCGATATGGCAGAGCG | 5’ primer for cloning *parC* of *E. coli* onto pET-SUMO |
| EcParC-*Sal I*-R | ATTGTCGACTTACTCTTCGCTATCACCGC | 3’ primer for cloning *parC* of *E. coli* onto pET-SUMO |
| PaParE-*Sac I* F | ATTGAGCTCATGGCTACTTACAACGCAGAC | 5’ primer for cloning *parE* of *P. aeruginosa* onto pET-SUMO |
| PaParE-*Hind III* R | ATTAAGCTTCAGACCAGCACCTCGGCCAG | 3’ primer for cloning *parE* of *P. aeruginosa* onto pET-SUMO |
| PfParE-U-*Bgl II*-F | ATTAGATCTATGGCCACTCCCAGCGCTAGC | 5’ primer for cloning the N-terminal *parE* of *P. fluorescens* onto pET-SUMO |
| PfParE-U-*Age I*-R | CAGGGCCAAACCGGTTTCC | 3’ primer for cloning the N-terminal *parE* of *P. fluorescens* onto pET-SUMO |
| PfParE-D-*Age I*-F | GGAAACCGGTTTGGCCCTG | 5’ primer for cloning the C-terminal *parE* of *P. fluorescens* onto pET-SUMO |
| PfParE-D-*Xho I*-R | ATTCTCGAGTCAGGCCAGCACCTCAGC | 3’ primer for cloning the C-terminal *parE* of *P. fluorescens* onto pET-SUMO |
| PfParE-*Xba I*-F | ATTTCTAGAGATGGCCACTCCCAGCGCTAG | 5’ primer for cloning *parE* of *P. fluorescens* onto pDB-His |
| PfParE-*Xho I*-F | ATTCTCGAGGGCCAGCACCTCAGCCAGG | 3’ primer for cloning *parE* of *P. fluorescens* onto pDB-His |
| SaGrlB-*BamH I*-F | ATTGGATCCATGAATAAACAAAATAATTATTCAGATG | 5’ primer for cloning *grlB* of *S. aureus* onto pET-SUMO |
| SaGrlB-*Sal I*-R | ATTGTCGACTAGATTTCCTCCTCATCAAATTG | 3’ primer for cloning *grlB* of *S. aureus* onto pET-SUMO |
| SpGrlB-*BamH I*-F | ATTGGATCCGTGTCAAAAAAGGAAATCAATATTAAC | 5’ primer for cloning *grlB* of *S. pneumoniae* onto pET-SUMO |
| SpGrlB-*Sal I*-R | ATTGTCGACTTAAAACACTGTCGCTTCTTCTAGC | 3’ primer for cloning *grlB* of *S. pneumoniae* onto pET-SUMO |
| YpParE-*Sac I* -F | ATTGAGCTCATGACTGAATCCAGCTATAACG | 5’ primer for cloning *parE* of *Y. pseudotuberculosis* onto pET-SUMO |
| YpParE-*Xho I*-R | ATTCTCGAGTCAGACCTCGATCTCAGCAG | 3’ primer for cloning *parE* of *Y. pseudotuberculosis* onto pET-SUMO |
| PfFic-1_H135A_-F | GGGGCTACGACGTTGATGTCGG | Primer for Fic-1 H135A mutation |
| PfFic-1_H135A_-R | CTTCCGCGAAGGCAATGGCC |  |
| PfFic-2_H179A_-F | GATGAATTCGAATTCGTAGTG | To mutate catalytic residue of Fic-2 |
| PfFic-2_H179A_-R | GCTCCCTTTGCAGACGGTAATGG |  |
| PfFic-2_E56G_-F | CACTCAACGTATTGTTTCCGATCGCCAGGGAAGC | To produce constitutive active form of Fic-2 |
| PfFic-2_E56G_-R | GCTTCCCTGGCGATCGGAAACAATACGTTGAGTG |  |
| PfFic-3_H205A_-F | AACAAGCGCCACTCGGTGATGACC | To mutate catalytic residue of Fic-3 |
| PfFic-3_H205A_-R | GCCCCCTTCCTCGATGGCAATGG |  |
| PfFic-3_E62G_-F | CGGTTCGATGCCCTCCGATCAGGTTCGAG | To produce constitutive active form of Fic-3 |
| PfFic-3_E62G_-R | CTCGAACCTGATCGGAGGGCATCGAACCG |  |
| Sa1560_E107G_-F | CAATCTCTTGTTTAGTACTAAATACATTTCCAATTTCATTAGTACTTTGTAATTCATCG | To produce constitutive active form of Sa1560 |
| Sa1560_E107G_-R | CGATGAATTACAAAGTACTAATGAAATTGGAAATGTATTTAGTACTAAACAAGAGATTG |  |
| Sa1560_H236A_-F | CCATTGCCATCATAAAAAGGAGCTATATATTCAAACAGATAATGACTAGCCAT | To mutate catalytic residue of Sa1560 |
| Sa1560_H236A_-R | ATGGCTAGTCATTATCTGTTTGAATATATAGCTCCTTTTTATGATGGCAATGG |  |
| Sp0496_E40G_-F | GTTTCTCAGCCAAAGATCCCATATACGCTGATGGC | To produce constitutive active form of Sp0496 |
| Sp0496_E40G_-R | GCCATCAGCGTATATGGGATCTTTGGCTGAGAAAC |  |
| Sp0496_E243G_-F | TGACCACCGAGCAAGAGATTTCCAAAAAAAGCTGTTAAAAACTC | To produce constitutive active form of Sp0496 |
| Sp0496_E243G_-R | GAGTTTTTAACAGCTTTTTTTGGAAATCTCTTGCTCGGTGGTCA |  |
| PA0574_E68G_-F | CATTCAGTTGCTCTCCCCCAATGGCTGAAGAGGTG | To create constitutive active form of PA057 |
| PA0574_E68G_-R | CACCTCTTCAGCCATTGGGGGAGAGCAACTGAATG |  |
| PfGyrB_Y111A_-F | ACCGCCGGAGACCTTGGCGGAGTTATCGTCGAAC | Primer for GyrB Y111A mutation of *P. fluorescens* |
| PfGyrB_Y111A_-R | GTTCGACGATAACTCCGCCAAGGTCTCCGGCGGT |  |
| PfParE_Y109A_-F | CAGACCGCCGGAGAACTGGGCGTTCTTGTTGGAAAACTTG | Primer for ParE Y109A mutation of *P. fluorescens* |
| PfParE_Y109A_-R | CAAGTTTTCCAACAAGAACGCCCAGTTCTCCGGCGGTCTG |  |
| EcGyrB_Y109F_-F | ATTTGACGATAACTCCTTTAAAGTGTCCGGCGGTC | Primer for GyrB Y109F mutation of *E. coli* |
| EcGyrB_Y109F_-R | GACCGCCGGACACTTTAAAGGAGTTATCGTCAAAT |  |
| PfParE_Y109A_-F | CAGACCGCCGGAGAACTGGGCGTTCTTGTTGGAAAACTTG | To mutate Tyrosine109 of ParE from *P. fluorescens* to Alanine |
| PfParE_Y109A_-R | CAAGTTTTCCAACAAGAACGCCCAGTTCTCCGGCGGTCTG |  |
| YpParE_Y106A_-F | GTGCAAACCACCAGAAAACTGAGCGTTCTTATTGGAGAATTTACCG | To mutate Tyrosine106 of ParE from *Y. pseudotuberculosis* to Alanine |
| YpParE_Y106A_-F | CGGTAAATTCTCCAATAAGAACGCTCAGTTTTCTGGTGGTTTGCAC |  |
| SaGrlB_Y109A_-F | TGAAGACCACCTGAAGTTTTAGCGCCGCCTTGTCCAAATTTAC | To mutate Tyrosine109 of GrlB from *S. aureus* to Alanine |
| SaGrlB_Y109A_-R | GTAAATTTGGACAAGGCGGCGCTAAAACTTCAGGTGGTCTTCA |  |
| EcParE_Y105F_-F | GCAGGCGGTAAATTCTCTAACAAAAATGCCCAGTTCTCTGGCG | To mutate Tyrosine105 of GrlB from *E. coli* to Alanine |
| EcParE_Y105F_-R | CGCCAGAGAACTGGGCATTTTTGTTAGAGAATTTACCGCCTGC |  |

Supplementary Table 3. Source data for Figure 6D

| Fic protein | Replication | LexA | LexA  background | RecA | RecA  background | ICDH | ICDH background |
| --- | --- | --- | --- | --- | --- | --- | --- |
| Fic-1 | rep1 | 185.20 | 216.22 | 146.57 | 221.83 | 157.42 | 220.63 |
|  | rep2 | 194.52 | 217.71 | 161.08 | 223.53 | 146.59 | 221.74 |
|  | rep3 | 202.83 | 217.50 | 160.51 | 224.89 | 150.33 | 221.22 |
| Fic-1_H135A_ | rep1 | 147.81 | 216.30 | 168.37 | 223.18 | 133.70 | 223.29 |
|  | rep2 | 187.37 | 216.87 | 173.78 | 224.28 | 124.22 | 222.03 |
|  | rep3 | 186.77 | 217.06 | 171.16 | 224.99 | 132.41 | 223.81 |
| Fic-2 | rep1 | 159.22 | 215.98 | 172.94 | 223.44 | 122.16 | 223.16 |
|  | rep2 | 183.47 | 217.25 | 163.67 | 224.02 | 115.64 | 220.84 |
|  | rep3 | 176.64 | 216.44 | 173.18 | 224.99 | 131.50 | 224.07 |
| Fic-2_E56G_ | rep1 | 186.98 | 216.82 | 149.90 | 222.03 | 141.41 | 222.42 |
|  | rep2 | 203.47 | 217.21 | 145.53 | 222.89 | 139.19 | 219.54 |
|  | rep3 | 201.33 | 216.30 | 153.53 | 224.93 | 141.55 | 223.60 |
| Fic-2_H179A_ | rep1 | 151.64 | 217.04 | 164.45 | 222.57 | 118.58 | 222.79 |
|  | rep2 | 187.01 | 215.86 | 167.41 | 224.05 | 113.84 | 220.64 |
|  | rep3 | 179.67 | 214.58 | 169.13 | 225.00 | 128.05 | 224.03 |
| Vector | rep1 | 156.79 | 216.93 | 169.49 | 220.23 | 129.85 | 223.30 |
|  | rep2 | 185.31 | 216.95 | 179.93 | 223.98 | 109.07 | 219.15 |
|  | rep3 | 189.05 | 215.52 | 168.87 | 224.94 | 131.61 | 224.09 |
| Ciprofloxacin | rep1 | 211.95 | 217.67 | 90.44 | 200.58 | 128.14 | 205.33 |
|  | rep2 | 213.04 | 217.13 | 74.08 | 207.41 | 129.05 | 200.35 |
|  | rep3 | 210.31 | 215.54 | 76.68 | 202.74 | 106.94 | 200.91 |
| PA1366 | rep1 | 173.07 | 216.50 | 155.12 | 223.04 | 139.50 | 222.39 |
|  | rep2 | 184.12 | 218.15 | 162.22 | 224.80 | 145.99 | 222.93 |
|  | rep3 | 188.97 | 217.91 | 160.56 | 224.93 | 143.15 | 221.92 |
| PA1366_H136A_ | rep1 | 144.71 | 216.09 | 161.21 | 223.88 | 137.15 | 224.23 |
|  | rep2 | 179.17 | 217.96 | 168.40 | 224.42 | 126.34 | 221.76 |
|  | rep3 | 179.93 | 217.98 | 155.27 | 224.63 | 119.94 | 222.02 |
| FicY | rep1 | 188.85 | 217.82 | 156.86 | 223.08 | 131.59 | 219.70 |
|  | rep2 | 193.83 | 216.52 | 130.37 | 221.57 | 120.70 | 223.11 |
|  | rep3 | 195.02 | 216.27 | 145.35 | 224.11 | 129.98 | 223.34 |
| FicY_H141A_ | rep1 | 173.26 | 217.41 | 158.47 | 222.24 | 122.79 | 217.86 |
|  | rep2 | 175.50 | 215.88 | 167.35 | 224.44 | 112.42 | 224.01 |
|  | rep3 | 179.71 | 215.66 | 167.67 | 224.78 | 133.82 | 224.05 |

### **References**

Engel, P., Goepfert, A., Stanger, F.V., Harms, A., Schmidt, A., Schirmer, T., et al. (2012). Adenylylation control by intra- or intermolecular active-site obstruction in Fic proteins. *Nature* 482(7383)**,** 107-110. doi: 10.1038/nature10729.

Guzman, L.-M., Belin, D., Carson, M.J., and Beckwith, J. (1995). Tight regulation, modulation, and high-level expression by vectors containing the arabinose PBAD promoter. *Journal of Bacteriology* 177(14)**,** 4121-4130. doi: 10.1128/jb.177.14.4121-4130.1995.

Hanahan, D. (1983). Studies on transformation of *Escherichia coli* with plasmids. *Journal of Molecular Biology* 166(4)**,** 557-580. doi: 10.1016/S0022-2836(83)80284-8.

Karimova, G., Pidoux, J., Ullmann, A., and Ladant, D. (1998). A bacterial two-hybrid system based on a reconstituted signal transduction pathway. *Proceedings of the National Academy of Sciences of the United States of America* 95(10)**,** 5752-5756. doi: 10.1073/pnas.95.10.5752.

Lu, C., Nakayasu, E.S., Zhang, L.-Q., and Luo, Z.-Q. (2016). Identification of Fic-1 as an enzyme that inhibits bacterial DNA replication by AMPylating GyrB, promoting filament formation. *Science Signaling* 9(412)**,** ra11. doi: 10.1126/scisignal.aad0446.

McCafferty, D.G., Lessard, I.A.D., and Walsh, C.T. (1997). Mutational analysis of potential zinc-binding residues in the active site of the enterococcal D-Ala-D-Ala dipeptidase VanX. *Biochemistry* 36(34)**,** 10498-10505. doi: 10.1021/bi970543u.

Wei, H.-L., and Zhang, L.-Q. (2006). Quorum-sensing system influences root colonization and biological control ability in *Pseudomonas fluorescens* 2P24. *Antonie van Leeuwenhoek* 89(2)**,** 267-280. doi: 10.1007/s10482-005-9028-8.
